# Sulfur-containing sustainable polymers: synthetic pathways, degradation mechanisms, and multifunctional applications

**DOI:** 10.1093/nsr/nwaf475

**Published:** 2025-11-03

**Authors:** Haiyang Song, Jiaolong Meng, Xuefeng Jiang

**Affiliations:** Hainan Institute of East China Normal University, Shanghai Key Laboratory of Green Chemistry and Chemical Processes, State Key Laboratory of Molecular & Process Engineering, School of Chemistry and Molecular Engineering, East China Normal University, Shanghai 200062, China; Hainan Institute of East China Normal University, Shanghai Key Laboratory of Green Chemistry and Chemical Processes, State Key Laboratory of Molecular & Process Engineering, School of Chemistry and Molecular Engineering, East China Normal University, Shanghai 200062, China; Hainan Institute of East China Normal University, Shanghai Key Laboratory of Green Chemistry and Chemical Processes, State Key Laboratory of Molecular & Process Engineering, School of Chemistry and Molecular Engineering, East China Normal University, Shanghai 200062, China; School of Chemistry and Chemical Engineering, Henan Normal University, Xinxiang 453007, China; State Key Laboratory of Organometallic Chemistry, Shanghai Institute of Organic Chemistry, Chinese Academy of Sciences, Shanghai 200032, China

**Keywords:** degradable sulfur-containing polymers, multifunctional applications, closed-loop recycling, dynamic covalent adaptability

## Abstract

Degradable sulfur-containing polymers leverage the controllable cleavage of thioester and thioether bonds under external stimuli, coupled with closed-loop recyclability, to significantly mitigate plastic pollution. The dynamic covalent adaptability of sulfur bonds within the polymer backbone, combined with the high refractive index arising from sulfur’s high polarizability, enables the integration of stimuli-responsive degradation and multifunctionality. Advanced polymerization techniques, such as ring-opening polymerization and reversible addition-fragmentation chain-transfer polymerization, permit precise control over the architecture of the backbone, yielding high-performance materials with tunable thermal, mechanical, and optical properties. These materials demonstrate significant potential in biomedicine, environmental remediation, and energy storage. Focusing on molecular design, degradation control, and functional diversification, this review systematically elucidates synthetic strategies, degradation mechanisms, and frontier applications, while providing perspectives on future developments.

## Teaser

This review highlights how degradable sulfur-containing polymers leverage dynamic covalent bonds to enable controllable degradation, closed-loop recycling, and multifunctional applications in biomedicine, environmental remediation, and energy storage.

## INTRODUCTION

**Figure 1.**
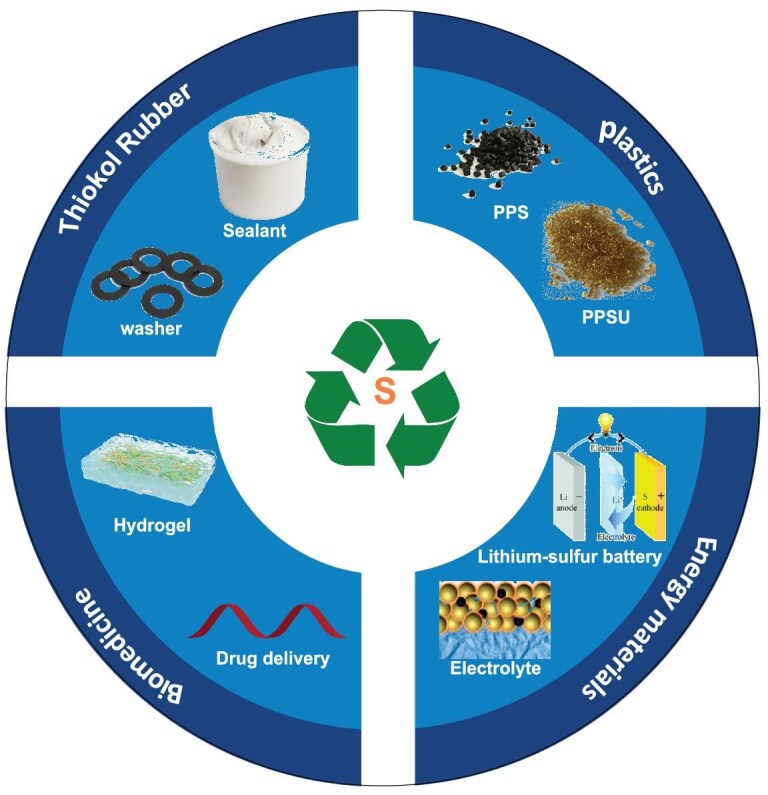
Multifunctional applications of sulfur-containing polymers.

Sulfur, as one of the earth’s most abundant elements, is widely distributed in fossil fuels, mineral salts, and biomolecules [1,2]. It finds extensive applications across biochemistry [3], pharmaceutical synthesis [4,5], and polymer materials science (Fig. 1) [6,7]. Sulfur’s unique electronic structure—characterized by the high polarizability of lone-pair electrons in its 3p orbitals and versatile redox capabilities across multiple oxidation states (−2 to +6) [8,9]—endows it with inherent advantages for constructing dynamic covalent bonds [10–12]. This is manifested through diverse responsive mechanisms in sulfur-based dynamic bonds: (1) disulfide bonds (–S–S–) [13,14]: redox responsiveness; (2) thiocarbonyl structures [15] (C=S in thionolactones): photolytic/thermal cleavage; (3) thioester bonds (C(=O)–S) [16,17]: hydrolytic controllability (the fundamental structural difference between thiolactones and thionolactones, which dictates their distinct degradation pathways, is summarized for clarity in Table 1). These properties enable sulfur-based dynamic materials to achieve reversible crosslinking and functional switching under thermal, photonic, or chemical stimuli [18]. Such intrinsic dynamism not only overcomes the non-recyclability limitation of traditional thermosets but also facilitates intelligent behaviors like self-healing [19] and shape memory [20].

**Table 1.**
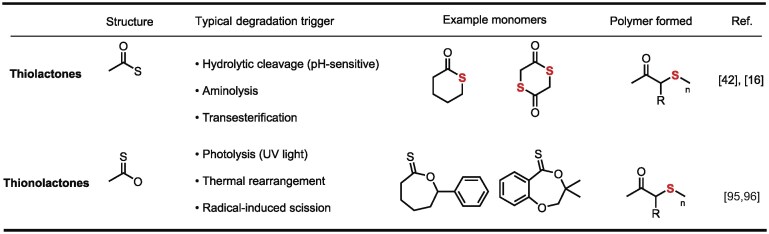
Structural distinction between thiolactones and thionolactones.

**Figure 2.**
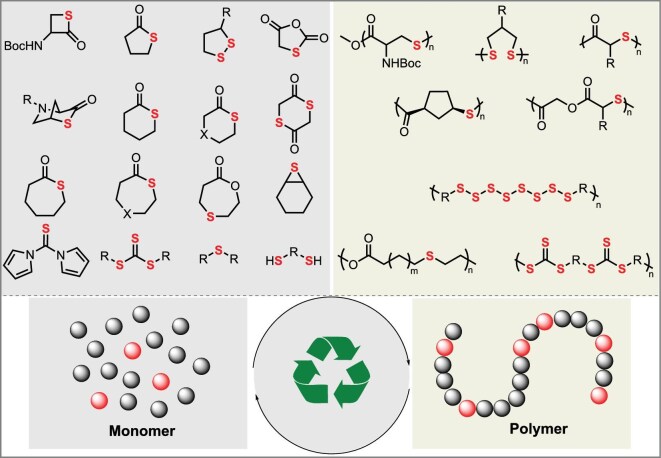
Representative monomers and polymers.

In recent years, sulfur-containing polymers designed with high-selectivity and high-purity sulfur-based monomers have rapidly advanced in the field of degradable materials (Fig. 2) [21]. These polymers can degrade into pristine monomers under specific stimuli, enabling a closed-loop recycling [22–28]. The recovered monomers can be efficiently repolymerized into materials with performance identical to the original, making them an ideal system for realizing a plastic ‘circular economy’. Their core advantages include: (1) significantly reducing pollution of aquatic and terrestrial ecosystems by polymer waste, thereby mitigating associated ecological damage, climate impacts, and public health risks [29]; (2) opening new pathways for valorizing sulfur, enhancing resource utilization efficiency [30]; (3) lowering regeneration costs through mild-condition reversibility and high monomer recovery value, thereby strengthening economic competitiveness of recycling systems. Additionally, sulfur-based polymers exhibit performance advantages surpassing traditional carbon/oxygen-based materials, such as high refractive index, inherent antimicrobial properties, and metal coordination capabilities. These attributes position sulfur-based polymers as a bridge between ‘green chemistry’ and ‘high-performance materials’ [31–34]. By converting sulfur by-product into functional polymers, they not only achieve resource circularity but also drive the transition of materials science towards a sustainable paradigm.

Sulfur chemistry offers a revolutionary paradigm for the design of recyclable polymers by leveraging its unique toolbox of dynamic bonds and potential for resource circulation. However, translating this chemical advantage into practical applications requires systematically addressing three core challenges: how to precisely synthesize sulfur-based networks that combine dynamicity with high performance; how to design efficient and green degradation pathways to achieve closed-loop recycling; and how to uncover the irreplaceable role of sulfur-based materials in diverse scenarios. This review will specifically focus on three dimensions—molecular synthesis, degradation mechanisms, and functional expansion—and provide an outlook on the development of this field.

## BUILDING SULFUR-CONTAINING POLYMER BACKBONES VIA ROP

**Figure 3.**
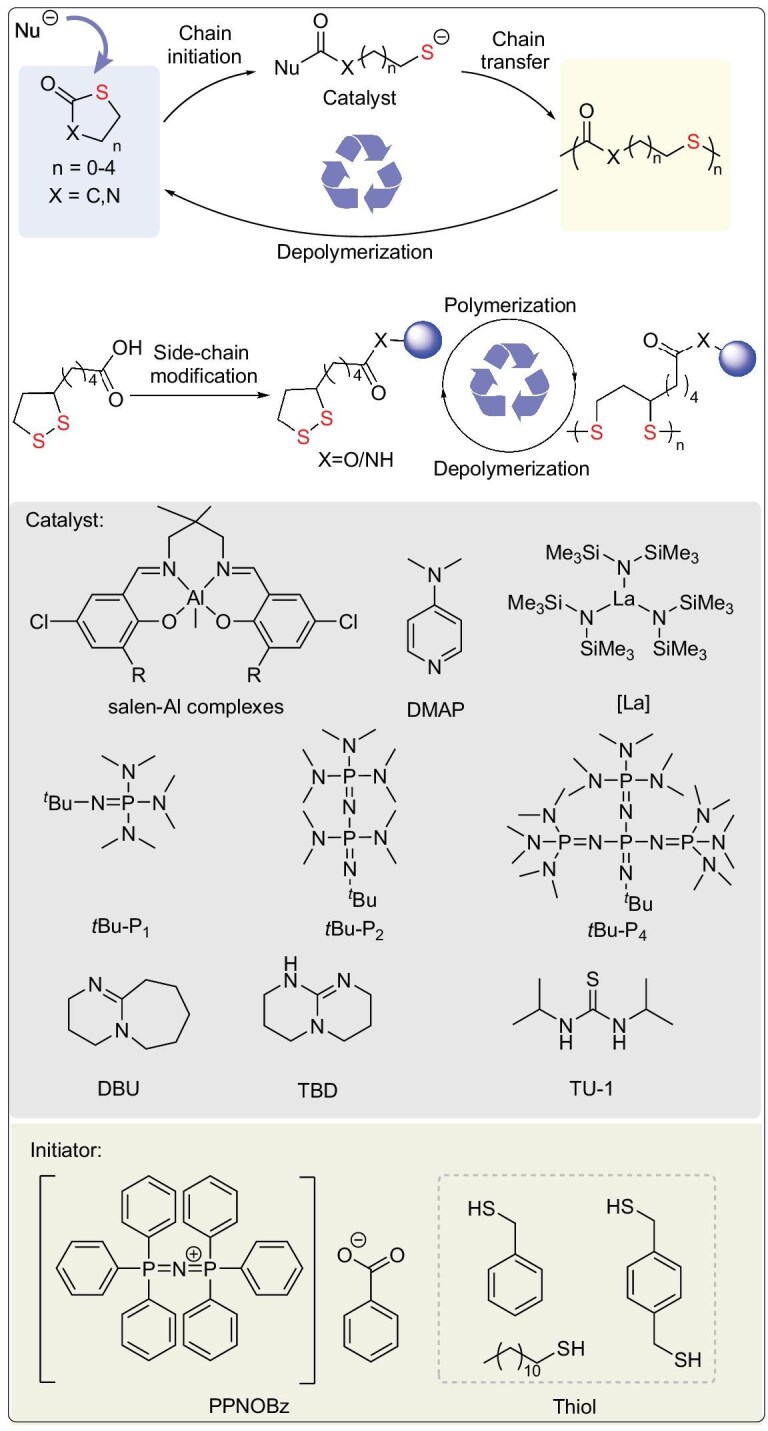
Thiolactones and cyclic disulfides: ROP and commonly used initiators/catalysts.

Ring-opening polymerization (ROP) [35], leveraging its core advantages of thermodynamic favorability, precise backbone sequence control, and efficient energy release from ring strain, serves as a powerful synthetic strategy for constructing sulfur-containing polymer backbones. This approach is particularly effective for two major monomer classes: thiolactones and strained cyclic disulfides (Fig. 3).

### The ROP of thiolactones

While traditional lactone ROP enables a closed-loop ‘monomer-polymer-monomer’ cycle, its high energy requirements constrain sustainable development. In contrast, thiolactone ROP introduces dynamic thioester linkages (C(=O)–S) (Table 2) [36,37], endowing the system with three transformative advantages: (1) reversible polymerization-depolymerization cycling near ambient temperatures, drastically reducing energy consumption and costs; (2) sulfur incorporation induces significant enhancements in infrared transmittance and other optical properties, alongside improved thermal stability, overcoming limitations of hydrocarbon polymers; (3) direct synthesis of high-molecular-weight polythioesters under mild conditions via nucleophilic initiators (amines/thiols/carboxylates)—a more eco-friendly approach than thioester condensation polymerization—providing sustainable pathways for biodegradable biomedical materials and optical devices.

**Table 2.**
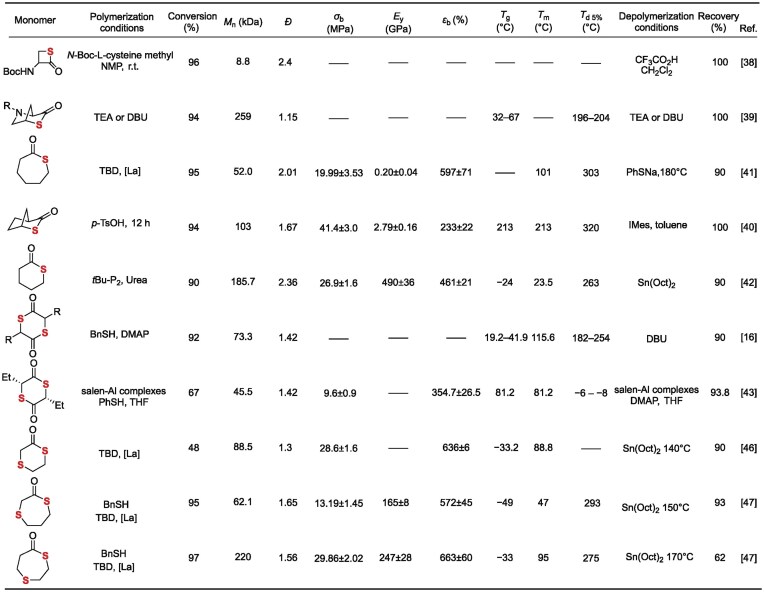
Selected thiolactone structures, polymerization conditions, monomer conversion, number average molecular weight (*M*_n_), polymer dispersity index (*Ð*), tensile strength (*σ*_b_), Young’s modulus (*E*_y_), elongation at break (*ε*_b_), glass transition temperature (*T*_g_), melting temperature (*T*_m_), temperature at 5% weight loss (*T*_d 5%_), depolymerization conditions, and monomer recovery for polyacetals based on thiolactones.

#### Amino acid-based thiolactone sulfur-containing polymers

**Figure 4.**
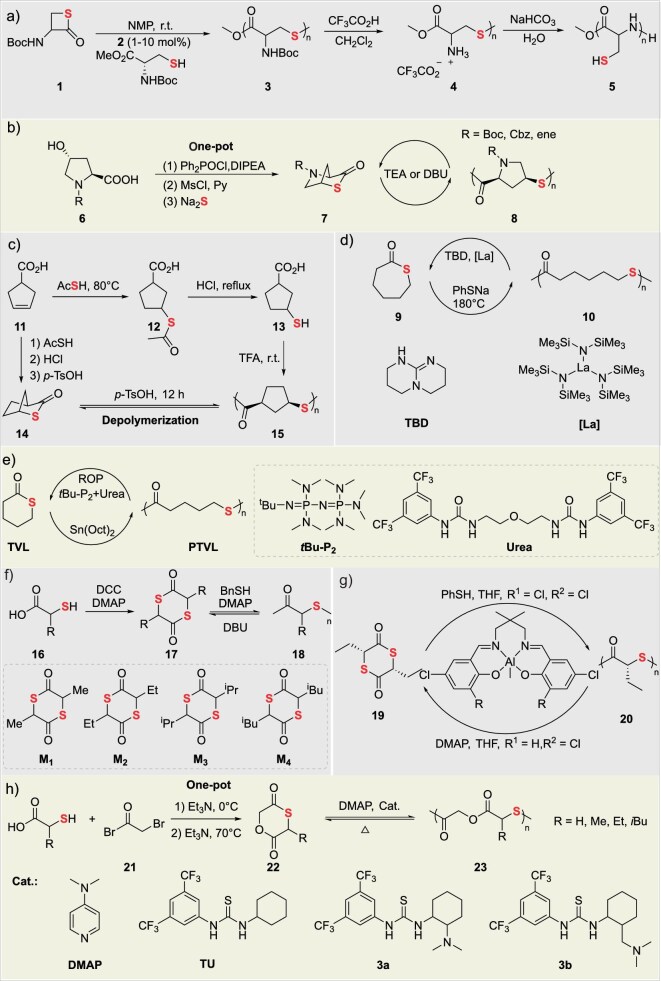
Degradable sulfur-containing polymers via thiolactone ROP: (a) thiolactone modification through nucleophilic substitution and deprotection. (b) One-pot synthesis of thiolactones from amino acid derivatives to sulfur heterocycles. (c) Sulfur heterocycle and polymer construction from small-ring thiolactones. (d) Macrocyclic thiolactone polymerization using base or organometallic catalysts. (e) Thiolactone TVL polymerization yielding PTVL. (f) Sulfur-containing linear molecules via thiolactone ring-opening. (g) Chiral sulfur compound synthesis from thiolactones and thiophenols. (h) Sulfur polymer preparation from thiocarboxylic acids and bromoketones.

Polythioesters obtained from amino acid-based thiolactone monomers have garnered extensive academic attention owing to their abundant renewable resources, amino acid substituents, and versatile functionalization. In 2016, Suzuki’s group addressed two key challenges—poor controllability in traditional *β*-thiolactone ROP and limited research on biodegradable polythioesters—by developing a thiol-mediated controlled ROP of *N*-Boc-cysteine-derived *β*-thiolactone (**1**) in *N*-methylpyrrolidone at room temperature (Fig. 4a) [38]. This system employed a unique C(=O)–S bond cleavage mechanism, where the thiol group acted as the propagating chain end, enabling precise molecular weight control (*M*_n_ = 2.9–8.8 kDa, *Đ* = 1.6–2.4) via initiator/monomer feed ratios. Subsequent trifluoroacetic acid-mediated Boc deprotection triggered intramolecular *S*-to-*N* acyl migration, converting the polythioester backbone into polycysteine with 49% yield. This process demonstrates topologically precise backbone reconstruction via dynamic covalent chemistry. In 2019, Lu’s group developed a biomass-derived *trans*-4-hydroxy-L-proline-derived (**6**) *N*-substituted *cis*-4-thia-L-proline thiolactone (N^R^-PTL) (**7**) monomer. Through base (TEA/DBU)-catalyzed controlled ROP in chloroform at 25°C, high-molecular-weight (*M*_n_ ≥259 kDa), narrow-dispersity (*Đ* <1.15) polythioesters (PTEs) were efficiently synthesized (Fig. 4b) [39]. The high ring strain of the bicyclic structure drives polymerization, while steric hindrance at the proline-proline junction and n→π* orbital interaction between urethane and thioester carbonyls suppress chain transfer, enabling precise control. These PTEs exhibit three key characteristics: (1) quantitative depolymerization to monomers in dilute alkaline solution (0.046 equiv. DBU, 50°C, 2 min), with 79% monomer recovery after thermolysis at 160°C; (2) tunable properties via >95% efficient side-chain functionalization (e.g. carboxylic acid grafting via click chemistry), yielding an adjustable glass transition temperature (*T*_g_ 32–67°C); (3) stability under base-free conditions (5 d at 50°C) and controllable degradability, supporting applications in optical plastics, self-immolative polymers, and biomaterials. By resolving the conflict between high polymerization reactivity and controlled depolymerization through molecular design, this work establishes a closed-loop recyclable platform.

#### Monothiolactone-derived sulfur-containing polymers

Compared to amino acid-derived monomers, thiolactones have emerged as a pivotal platform for high-performance, closed-loop recyclable polythioesters due to their structural simplicity, tunable alkyl chain length/branching, and resulting precise control over crystallinity, mechanical strength, and thermal properties. In 2020, Chen’s group achieved unprecedented control over poly(butylene thiolactone) (PBTL) (**9**) tacticity (isotactic to atactic) and topology (linear vs cyclic) through a bridged bicyclic thiolactone monomer (2-thiabicyclo[2.2.1]heptan-3-one) and stereoselective ROP with organic base/metal catalysts. This approach challenged the conventional paradigm correlating stereoregularity with crystallinity (Fig. 4c) [40]. The resulting PBTL exhibits engineering plastic-grade performance: (1) Young’s modulus is 2.79 GPa, (2) tensile strength is 49 MPa, (3) elongation at break is >200%, (4) thermal decomposition temperature (*T*_d 5%_) is 328°C. Notably, it enables >90% monomer recovery via mild catalytic depolymerization (100°C) or room-temperature solution processes. Repolymerization of recycled monomers yields materials with identical properties to virgin PBTL, validating closed-loop feasibility.

In 2025, Li’s group utilized *δ*-thiovalerolactone (*δ*TVL) monomer to achieve crystallization/precipitation-driven non-equilibrium ROP using a phosphazene base (*t*BuP_2_)/bisurea binary catalyst (Fig. 4d) [41]. In tetrahydrofuran (THF) with high monomer concentration (8 mol L^−1^), the continuous crystallization and precipitation of poly(*δ*-thiovalerolactone) (PTVL) overcame *δ*TVL’s thermodynamic equilibrium limitations, yielding high-molecular-weight PTVL (*M*_n_ ≥185.7 kDa)—the first report of such an achievement. The PTVL exhibits exceptional properties including a high melting point (125°C), tensile strength of 26.9 MPa, elongation at break of 461%, and an oxygen permeability as low as 0.175 Barrer, comparable to commodity plastics. Catalyst-free bulk depolymerization at 200°C under reduced pressure (or with 0.5 mol% Sn(Oct)_2_) recovers *δ*TVL monomer in >95% yield and >99.5% purity. Notably, *δ*TVL can be selectively recovered from mixed plastic waste (e.g. PET/PP) via simple distillation (90% yield, >99.5% purity) without pre-sorting. In 2025, Li’s group synthesized thiocaprolactone (tCL) via sulfur substitution and prepared poly(thiocaprolactone) [P(tCL)] through ROP (Fig. 4e) [42]. This material exhibits multiple high-performance properties, including excellent thermal stability (*T*_d 5%_ = 273°C), mechanical properties close to low-density polyethylene with a tensile strength (*σ_b_* = 19.99% ± 3.53 MPa), and elongation at break (*ε_b_* = 597% ± 71%). Quantitative depolymerization (90% yield) was achieved in 2 h at 180°C using PhSNa catalyst, yield >99% pure tCL. Notably, tCL was selectively recovered from mixed plastic waste (PP/HDPE/PET) via vacuum distillation without separation, offering a scalable solution for recyclable food packaging.

#### Dithiolactone-derived sulfur-containing polymers

Dithiolactones have emerged as promising platforms for high-performance sustainable plastics due to their low ring strain energy, high polymerizability, and chemical recyclability. Stereoretentive polymerization enabled by nucleophilic catalysts produces polythioesters with enhanced thermomechanical properties. In 2021, Tao’s group pioneered *α*-amino acid-derived dithiolactone monomers (**17**) as lactone alternatives. Through 4-(*N,N*-dimethylamino) pyridine (DMAP)-catalyzed ROP in nonpolar solvents at 25°C, controlled polymerization yielded polythioesters with *M*_n_ ≤100.5 kDa (Fig. 4f) [16]. These materials achieved 98% monomer recovery within 2 min at 25°C under DBU catalysis and could be isolated in >95% yield via distillation. Despite their atactic configuration, the polymers exhibited high crystallinity due to side-chain group interactions, along with excellent thermal stability. Crucially, enhanced mechanical properties correlated with molecular weight: **PM3** (*M*_n_ = 100.5 kDa) demonstrated a tensile strength of 21.3 MPa and elongation at break of 140.1%—representing a 37-fold increase in ductility compared to its low-*M_w_* counterpart. Based on the above research, in 2023, the research group engineered aluminum complexes guided by hard/soft acid-base (HSAB) theory for stereoretentive ROP of dithiolactones (Fig. 4g) [43]. Precise modulation of ligand electronics and scaffold flexibility suppressed sulfur-induced catalyst poisoning and racemization. Using benzyl mercaptan as the initiator, they prepared highly isotactic polythioesters with a molar mass of up to 45.5 kDa and a tacticity (*P*_m_) of 0.92, breaking through the limitations of traditional metal catalysts in sulfur-containing systems. The obtained isotactic polythioesters exhibit excellent comprehensive properties: the melting temperature of a single component reaches 83.4°C; its tensile strength (12.0 MPa) and elongation at break (100.9%) are comparable to those of petroleum-based low-density polyethylene (LDPE). Additionally, the reaction innovatively employs an aluminum precatalyst-mediated low-temperature depolymerization pathway, achieving a monomer recovery rate of up to 90% at 25°C while maintaining high stereoselectivity. In the same year, the research group again proposed a single-atom oxygen-by-sulfur substitution strategy. This strategy reduced the ring strain of the six-membered dilactone from 16.0 kcal·mol^−1^ to 9.1 kcal·mol^−1^ and enabled the design and synthesis of a series of monothiodilactone monomers (Fig. 4h) [44]. Using organic bases (TBD, DBU) co-catalyzed with thiourea-based catalysts, regioselective ROP of these monomers was conducted at room temperature. Selective cleavage of the acyl-S bond led to the formation of an alternating thioether-ester backbone, demonstrating regioselective ROP mediated by an aminophenyl thiourea catalyst. The resulting polymers achieved a *M*_n_ of ∼120 kDa and a *Đ* <1.1. The synthesized polythioesters exhibited both toughness comparable to LDPE and high thermal stability, while achieving 95% recovery of high-purity monomer via bulk sublimation. This work offers a new approach towards replacing conventional plastics.

#### Thioether-thioester heterocycle-derived sulfur-containing polymers

**Figure 5.**
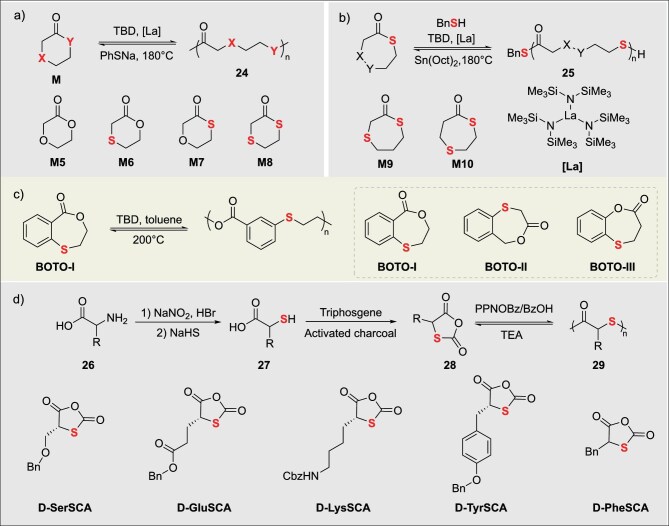
(a) Tuning polymerization behavior of ether-ester monomers via heteroatom substitution. (b) Chemically recyclable sulfur-containing polymers from ROP of seven-membered thiolactones. (c) Elevated-temperature ROP and structural elucidation of BOTO thiolactones. (d) Construction and derivatization reactions of amino acid-derived thiolactones.

Thioether-esters have attracted extensive attention in the fields of sustainable polymer materials and chemical recycling due to their advantages, such as tunable polymerization performance, high chemical recyclability, excellent thermal stability, and mechanical properties comparable to polyethylene [45]. In 2024, Wang’s group systematically elucidated the mechanism of the precise regulation of six-membered thioether-ester monomers via organobase-catalyzed ROP. Their study demonstrated that thio-substitution (**M**) enhances the *α*-H acidity of monomers and the nucleophilicity of thiolate chain ends, significantly accelerating polymerization kinetics (Fig. 5a) [46]. Furthermore, the differences in substitution sites were shown to dominate the diversity of material properties, including *T*_g_, melting point, crystallization behavior, and thermal stability. Remarkably, thioether-thioester polymers were found to exhibit mechanical properties comparable to HDPE (tensile strength of 28.6 MPa, elongation at break of 636%) and achieved breakthrough ultra-fast depolymerization (>99% conversion) catalyzed at room temperature within 1 min, as well as thermal recycling with yields exceeding 90% at 140°C. In 2025, Zhu’s group addressed the challenge of balancing chemical recyclability and performance in sulfur-containing polymers (Fig. 5b) [47]. They designed and synthesized two seven-membered thiolactone monomers (**M9** and **M10**) through a differential sulfur-atom embedding strategy. Using the organic base TBD as the catalyst at room temperature, they achieved efficient ROP, affording high-molecular-weight poly(thioether-thioester)s [**PM9**: *M*_n_ up to 62.1 kDa; **PM10**: *M*_n_ up to 256 kDa]. These polymers were found to exhibit high thermal stability (*T*_d 5%_ = 293°C), excellent mechanical properties (tensile strength, *σ_b_* ≈ 30 MPa; elongation at break, *ε*_b_ ≈660%), and good air stability. In terms of chemical recyclability, **PM9** undergoes thermal depolymerization at 150°C in the presence of 1 mol% Sn(Oct)_2_ as the catalyst, achieving a monomer recovery yield of 93%. The recycled monomers could be repolymerized to conversions ≥92%, forming a chemical closed loop. Moreover, the polymers were demonstrated to possess novel utility for the selective adsorption and recovery of Au^3+^. This work provides a new strategy for next-generation high-performance sustainable sulfur-containing polymers that integrate recyclability and functionality.

#### Other types of thiolactones as monomers

Aromatic and heterocyclic functional monomers endow polymers with unique thermal stability, optical properties, or functional response capabilities by introducing aromatic rings or sulfur-containing heterocyclic structures into the thiolactone backbone, breaking through the performance limitations of traditional aliphatic polythioesters. In 2021, Li’s group designed and synthesized three benzothiolactone isomers (BOTO-I/II/III), and prepared high-performance semi-aromatic polythioesters via organocatalytic ROP (Fig. 5c) [48]. Among them, P(BOTO-I) exhibited excellent comprehensive properties, with a *T*_m_ of 146°C and impact toughness exceeding that of polylactic acid (PLA). In terms of chemical recyclability, a novel bulk thermal depolymerization-sublimation method was employed, achieving highly selective monomer recovery yields exceeding 93% under catalysis by Sn(Oct)_2_. The recycled monomers could be efficiently repolymerized to high conversion, successfully establishing a new sustainable plastic system that integrates mechanical strength with closed-loop recycling capability.

In 2021, Tao’s group developed a class of amino acid-based thio-carboxyanhydride monomers and achieved ultra-fast, highly selective ROP of polythioesters using a PPNOBz/benzoic acid co-catalytic system (Fig. 5d) [17]. This approach overcame the challenges of uncontrollable transthioesterification and prevalent side reactions in traditional thiolactone polymerization. The system enabled complete monomer conversion within 1–2 min at room temperature in open air, affording polythioesters with precise molecular weights (*M*_n_ = 5–96 kDa), narrow dispersity (*Đ* <1.3), retained stereoregularity, and diverse side-chain functionalities. The polythioesters were efficiently depolymerized under triethylamine catalysis with monomer recovery yields exceeding 99%, and block copolymers were successfully synthesized. Density functional theory (DFT) studies revealed that the activation energy for chain propagation (7.9 kcal mol^−1^) was significantly lower than that for side reactions (transthioesterification: 16.0 kcal mol^−1^; backbiting: 14.5 kcal mol^−1^), and that CO_2_ release drives the thermodynamic favorability of ROP. This work establishes a theoretical and experimental foundation for the application of polythioesters in optical materials, recyclable engineering plastics, and related fields.

#### Summary

In summary, through the precise embedding of dynamic thioester bonds, ROP of thiolactones has pushed the ‘degradability’ and ‘functional programmability’ of sulfur-containing polymer backbones to new heights. This approach enables thermodynamically driven, mild polymerization, achieving not only reduced energy consumption compared with traditional lactone ROP, but also infrared transparency and increased *T*_g_ via the lone pair electron effect of sulfur atoms. Thus, it offers an irreplaceable material platform for bioabsorbable devices and infrared optical lenses. To advance the field beyond these current limitations, future research efforts should be directed towards the following priorities:

1. Addressing kinetic hysteresis in macrocyclic monomers through the development of novel catalytic systems that lower the activation barrier for ring-opening, thereby expanding the range of readily polymerizable macrocyclic thiolactones.
2. Overcoming stereocontrol limitations via innovative monomer engineering strategies, such as designing monomers with tailored chiral centers or steric hindrance, to achieve precise tacticity and enhance crystallinity and mechanical properties.
3. Evaluating and mitigating the potential toxicity of degradation products by establishing comprehensive toxicological profiling protocols early in the material design process, ensuring the biological safety of applications in biomedicine and environmental matrices.

### The ROP of cyclic disulfides

Following the ROP of thiolactones, cyclic disulfides have opened up dual-track pathways: ‘reversible rearrangement’ and ‘stimuli-responsive depolymerization’. This capability leverages the dynamic nature of their highly ring-strained disulfide bonds (–S–S–), characterized by a pentatomic ring bond angle ∠S–S–S ≈ 88° [6], enabling novel construction of sulfur-containing polymer backbones. Compared to the relatively static nature of thioester bonds, the –S–S– bonds activated during ROP possess both low bond dissociation energy (268 kJ mol^−1^) and high entropy change for isomerization (*ΔS* >40 J mol^−1^ K^−1^). These characteristics impart distinct advantages such as dynamic reversibility, functional integrability, closed-loop recyclability, and thermal reprocessability to the materials (Table 3). Consequently, this system demonstrates unique value for applications in adaptive sealing, recyclable plastics, and biocompatible stealth devices.

**Table 3.**
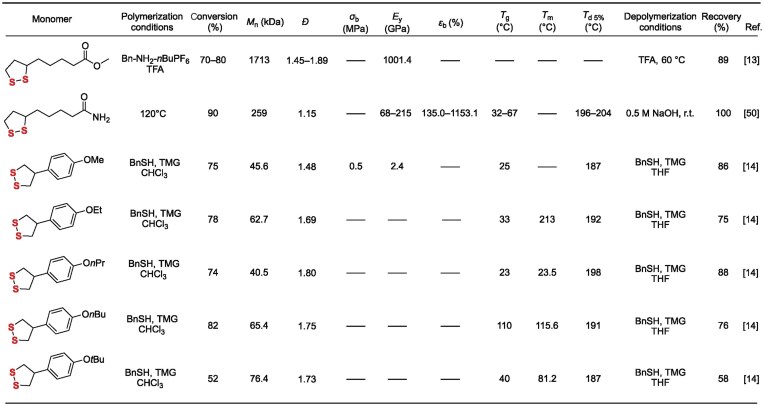
Selected cyclic disulfides' structures, polymerization conditions, monomer conversion, number average *M*_n_, polymer dispersity index (*Ð*), tensile strength (*σ*_b_), Young’s modulus (*E*_y_), elongation at break (*ε*_b_), glass transition temperature (*T*_g_), melting temperature (*T*_m_), temperature at 5% weight loss (*T*_d 5%_), depolymerization conditions, and monomer recovery for polyacetals based on cyclic disulfides.

**Table 4.** RAFT polymerization for sulfur-containing polymers: advantages, limitations, and design rules.

Aspect	Summary	Ref.
Key advantages	• **Precise control**: Enables the synthesis of polymers with predetermined molecular weights, narrow dispersity, and complex architectures. • **High functional group tolerance**: Compatible with various sulfur-containing functional groups (thioethers, thioesters, disulfides) without significant interference. • **Broad applicability**: Suitable for a wide range of vinyl monomers (acrylates, methacrylates, styrenics, etc.). • **End-group retention**: The thiocarbonylthio end-group allows for post-polymerization modification and chain extension.	[66,67]
Current limitations	• **Monomer scope**: Less effective for monomers with low propagation rates or those prone to side reactions. • **Potential side reactions**: Degradation or interference may occur under strong nucleophiles or harsh conditions due to the sensitivity of the RAFT agent. • **Degradation complexity**: After backbone degradation, persistent RAFT end-groups may remain, complicating product analysis. • **Purification**: Removing the characteristic color or odor associated with the RAFT agent after the reaction can be challenging.	[69,70]
Chain transfer agent (CTA)/Initiator selection guidelines	•**Matching principle**: The activity of the CTA should match the reactivity of the monomer. •**Avoid incompatible functional groups**: The initiator should not contain functional groups that can react with sulfur species or the RAFT agent. •**Consider degradation goals**: If complete degradation is desired, RAFT agents whose cleavage products are easy to remove should be selected. •**Steric effects**: CTAs with good leaving group ability should be chosen for synthesizing high-molecular-weight polymers.	[66,67] [69,70]

#### Sulfur-containing polymers derived from cyclic disulfides with varied side chains

**Figure 6.**
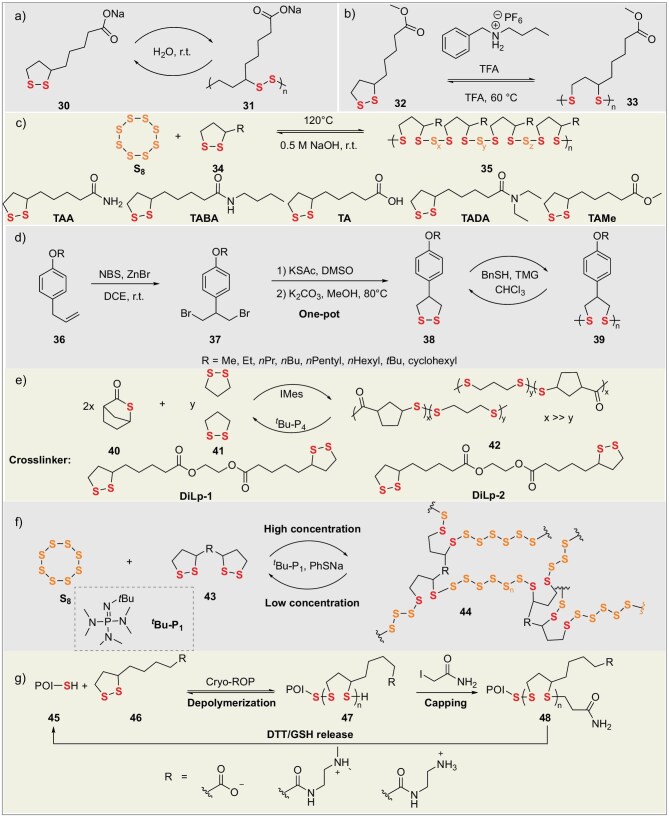
Chemical processes in ROP of cyclic disulfides: (a) water-promoted conversion and degradation. (b) Acid-catalyzed conversion and degradation. (c) S_8_ participated polymerization. (d) Substitution-induced cyclization and thio-ring modification. (e) Crosslinker applications. (f) Concentration-modulated polymerization. (g) Drug release-functionalized.

In 2019, Qu’s group overcame the disordered self-assembly limitation of natural thioctic acid (TA) via an innovative strategy. Deprotonating TA yielded amphiphilic sodium thioctate (ST) (**30**), enabling precise dynamic covalent ROP and evaporation-induced interfacial self-assembly (EIISA) (Fig. 6a) [49]. Driven by concentration gradients at the gas-liquid-solid interface, ST self-assembled into a highly ordered, long-range layered network. The resulting material exhibited key dynamic functions: (1) a dry Young’s modulus of 168.8 MPa, with adsorbed ‘structural water’ enhancing ductility under humidity; (2) complete scratch healing within 12 h (>50% humidity) and humidity gradient-driven reversible bending; (3) full degradation to ST monomers in water within 20 min at RT, with complete mechanical property recovery after EIISA regeneration. This work pioneers a pathway to low-cost, structurally ordered dynamic materials for applications like flexible sensors, humidity-responsive actuators, and recyclable polymers. In 2023, the same group developed an acid-catalyzed cationic ROP strategy mediated by the disulfide bond. Employing trifluoroacetic acid (TFA) in combination with hexafluorophosphate salts, protonation of the disulfide bond forms sulfonium cation active centers, enabling ultra-fast synthesis and efficient recycling of poly(disulfide)s (Fig. 6b) [13]. Under ambient, open-air conditions, polymerization reaches completion within minutes, achieving monomer conversion exceeding 80%. The resulting poly(disulfide)s (**33**) exhibit ultra-high molecular weights (*M*_n_ >1000 kDa, *Đ* = 1.6–1.9), tomeric properties (tensile modulus: 1001.4 kPa, elongation at break: >600%), and self-healing capability (95% healing efficiency at 40°C for 12 h). The materials demonstrate high absorption efficiency (>99%) for Au^3+^ ions. Quantitative depolymerization is achieved within 5 min under strong acid catalysis, yielding monomer recovery rates >89%. Key properties are retained above 90% after three recycling cycles. Building upon this research theme, the group developed a catalyst/solvent-free copolymerization strategy using cyclic disulfides (e.g. TAA, TAH derivatives) and S_8_. Radical ring-opening copolymerization (RROP) (Fig. 6c) [50], driven by ring strain and modulated by side-group H-bonding, yielded copolymers with up to 70 wt% sulfur. Young’s modulus was tunable (0.9–949 MPa). Poly(S_8_-TAH) fibers exhibited high strength (21.1 MPa) and toughness (9.4 MJ m^−3^). Dynamic H-bonds enabled room-temperature self-healing. The disulfide backbone allowed complete alkaline depolymerization to monomers, outperforming conventional polysulfides. Exceptional adhesion was achieved: lap-shear strength >10 MPa (steel/glass) and peel energy 5.36 kN m^−1^ for poly(S_8_-TAA/TAH). This integrated dynamic covalent/supramolecular approach addresses polysulfide challenges, establishing pathways for degradable thermoplastics and green adhesives.

In 2025, Liu’s group synthesized 4-aryl-1,2-dithiolane monomers (**38**) via gram-scale, one-pot NBS/ZnBr_2_-catalyzed bromination/cyclization of phenylpropanoid derivatives (Fig. 6d) [14]. Benzyl mercaptan/TMG-initiated anionic ROP yielded aryl-substituted poly(disulfide)s, exhibiting 21 times faster polymerization and higher thermal stability than unsubstituted analogs. Aryl side chains enhanced optical/mechanical properties. Recyclability was demonstrated: 80% monomer recovery within 1 h (25°C, 1 mol% thiolate catalyst), with repolymerization affording controllable-MW polymers. Unlike soft aliphatic poly(disulfide)s, the rigid aryl structure enables sustainable high-performance recyclable photoresists and high-refractive-index optical coatings.

#### Dynamic covalent cross-linked networks based on cyclic disulfides

In 2024, Chen’s group developed a copolymerization-crosslinking strategy between bicyclic thioester (BTL) (**40**) and a bifunctional dithiolane monomer (DiLp) (**41**). Utilizing anionic ROP catalyzed by IMes (1,3-bis(2,4,6-trimethylphenyl) imidazol-2-ylidene), they constructed a dynamically crosslinked network with semi-crystalline domains, achieving precise tuning of crystallinity and melting transition temperature (Fig. 6e) [51]. The material was fully depolymerized to BTL monomer within 1 h in solution or in the bulk state, catalyzed by 0.2 mol% phosphazene base *t*Bu-P_4_, in yields up to 95%. The regenerated monomer could be repolymerized to afford the vitrimer with virgin-like properties. This polymer exhibited unique characteristics: its semi-crystalline structure suppressed creep by restricting chain mobility, while imparting high stiffness (modulus: 0.72 GPa), high ductility (elongation at break: 396%), excellent toughness, and thermal stability.

In 2024, Qu’s group developed a closed-loop recyclable sulfur-based copolymer. This work employed cyclic disulfides (**43**) as replacements for diene crosslinkers, enabling efficient copolymerization of S_8_ with cyclic disulfides via room-temperature anionic ring-opening copolymerization (Fig. 6f) [52]. The method constructed a covalent adaptable network (CAN) with dynamic disulfide bonds (–S–S–) as crosslinks and polysulfide segments (–S_n_–) as the backbone, featuring tunable sulfur content (40–70 wt%). The polymer exhibited a tensile strength of up to 6.1 MPa, elongation at break >3000%, and near-infrared transmittance >80%, demonstrating potential for infrared optical applications. Under mild conditions (60°C), disulfide exchange mediated by thiolate anions triggered network cleavage, leading to depolymerization. This enabled recovery of cyclic disulfide monomer and S_8_ crystals in 85% yield with 95% purity. Remarkably, repolymerization yielded materials with properties comparable to the original.

In 2021, Li’s group developed a crosslinked network featuring an entirely disulfide-bonded backbone. This network was formed via ROP of azobenzene-containing cyclic monomers and bifunctional crosslinkers [53], catalyzed by TBD (1,5,7-triazabicyclo[4.4.0]dec-5-ene) and initiated by 1,6-hexanedithiol. The material integrates three dynamic features: (1) thermally triggered disulfide exchange enabling self-healing and reshaping; (2) catalytic depolymerization achieving high-efficiency monomer recovery (>90%); (3) azobenzene photoisomerization driving controllable shape changes. This work addresses the significant challenge of recyclability in traditional liquid crystal networks.

In 2022, Qu and Feringa’s group developed the first closed-loop recyclable crosslinked polymer network based on an orthogonal dynamic covalent chemistry (DCC) strategy [54]. This material employed acylhydrazine-functionalized dithiolane monomers as starting materials, achieving simultaneous main-chain disulfide polymerization and dynamic acylhydrazone crosslinking via solvent-free bulk polymerization. Linear polymers spontaneously depolymerized at room temperature in polar solvents, recovering monomers in 78% yield. The covalently crosslinked network required nucleophilic reagents to trigger acylhydrazone cleavage for orthogonal de-crosslinking, ultimately recovering virgin-quality monomers in 66% yield. The material exhibited significantly enhanced mechanical properties—the addition of just 2% crosslinker led to a 60-fold increase in modulus and a 4.7-fold increase in toughness (compared to the linear polymer)—while retaining thermal healability at 140°C.

#### Protein-polymer conjugates based on cyclic disulfides

In protein-polymer conjugate synthesis, traditional grafting-from strategies face limitations due to the need for pre-installed initiators on proteins and non-degradable products. In 2020, Lu’s group utilized cryogenic ROP technology to achieve, for the first time, the efficient *in situ* synthesis of reversibly degradable protein-polydisulfide conjugates (**47**) directly from native proteins (Fig. 6g) [55]. This method utilizes accessible cysteine residues as endogenous initiators to trigger ROP of 1,2-dithiolane monomers at −30°C. The conjugates release native protein quantitatively within 10 min upon mild reducing agent treatment. Mass spectrometry confirmed structural identity of released TEV protease-Cys-EGFP (**48**) fusion protein to the native form, while enzymatic assays verified full retention of activity. Incorporation of cationic comonomers during ROP imparts positive charges, endowing conjugates with efficient cell-penetrating capability and significantly enhanced cellular uptake versus free proteins. Applicable to sulfhydryl-containing proteins (e.g. BSA, DHFR), this strategy enables dynamic protein-of-interest (POI) functionalization and provides a versatile platform for: (1) cytosolic delivery of protein therapeutics; (2) protein function switching; (3) traceless protein purification. This technique requires no protein pre-modification and is compatible with *in situ* functionalization of native proteins.

#### Summary

Cyclic disulfides offer unique advantages for designing intrinsically dynamic smart materials through reversible ROP mediated by dynamic disulfide bonds. They leverage the inherent strength of covalent disulfide bonds coupled with their reversible exchange capability, which endows materials with room-temperature self-healing, stimuli-responsiveness (e.g. to light, heat, or reducing agents), and closed-loop recyclability. However, challenges remain, including limited thermal stability, difficulties in achieving precise control over polymerization, optimization of mechanical properties, and the development of efficient, eco-friendly processing and recycling technologies. Future efforts should focus on integrating main-chain engineering (e.g. diselenide incorporation) with advanced manufacturing techniques (e.g. three-dimensional printing) to expand the applications of these materials in biomimetic systems, recyclable electronics, and biomedicine.

### The ROP of cyclic dithioacetal

In 2023, Yang’s group developed a chemically recyclable polymer platform based on dynamic dithioacetal bonds. Linear polydithioacetals (PDTAs) were synthesized via acid-catalyzed step-growth polymerization [56]. Under dilute conditions, these polymers undergo entropy-driven ring-closing depolymerization to form macrocyclic monomers, which are efficiently regenerated into polymers via entropy-driven ROP in concentrated solutions, achieving closed-loop cycling with monomer conversion rates up to 95%. Crosslinked PDTA networks combine thermal reprocessability—enabled by low-activation-energy stress relaxation—with chemical recyclability.

In 2025, the same group developed fully chemically recyclable polydithioacetal materials. The researchers synthesized linear polymers via acid-catalyzed step-growth polymerization of aromatic/aliphatic dithiols and benzaldehyde monomers, and employed entropy-driven ROP to efficiently repolymerize macrocyclic monomers [57]. By varying monomer ratios, the *T*_g_ was tuned from −2 to 90°C, with significantly enhanced mechanical properties: elongation at break reached 387%, and tensile strength matched commercial low-density polyethylene (e.g. ∼20 MPa). The materials exhibited exceptional thermal stability, with *T*_d 5%_ up to 297°C. Polymers were completely degraded via ring-closing depolymerization into recyclable macrocyclic monomers under dilute conditions, enabling efficient monomer-to-polymer reconversion. The study further demonstrated benzaldehyde’s role in achieving high *M*_n_ (171 kDa), providing a strategy for sustainable alternatives to commodity plastics.

### Multifunctional applications

The application value of sulfur-containing polymers synthesized via the ROP strategy is closely linked to their precise molecular structures. Polythioesters obtained from amino acid-derived thiolactones show potential in biodegradable tissue engineering scaffolds and smart drug delivery systems due to their good biocompatibility and stimulus responsiveness [38,39]. Polysulfides based on mono/di-thiolactones, thanks to their excellent mechanical properties (such as high tensile strength and toughness) and efficient monomer ring-closing recycling capability, have become high-performance recyclable plastics that can replace traditional polyolefins [40,42]. Furthermore, networks containing dynamic disulfide bonds and aromatic polythioesters, leveraging their reconfigurability and high refractive index, respectively, hold a place in fields such as adaptive sealing materials, self-healing coatings, and high-refractive-index optical devices [13,48,51].

## SYNTHESES OF SULFUR-CONTAINING POLYMERS BY RADICAL POLYMERIZATION

**Figure 7.**
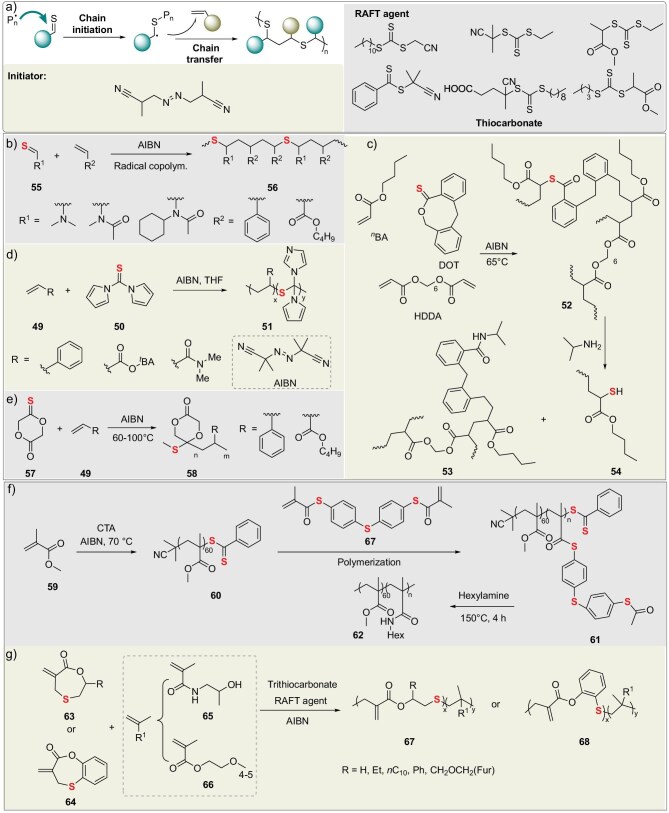
(a) Free radical polymerization: catalytic processes and commonly used initiators/catalysts. (b) Radical copolymerization of thio-functional vinyl monomers. (c) Radical polymerization of thioester monomers with post-polymerization derivatization. (d) Radical polymerization of sulfur-containing heterocyclic monomers. (e) Radical ring-opening polymerization (RROP) of thiolactones with vinyl monomers. (f) Degradable thioester core-crosslinked star-shaped polymers. (g) RAFT copolymerization of sulfur-containing cyclic monomers and vinyl monomers.

Precise control of the molecular and topological structures of sulfur-containing polymers is essential for achieving predictable degradation behavior. Controlled radical polymerization [58–60] provides powerful synthetic tools to accomplish this objective (Fig. 7a). This chapter elaborates on controlled radical polymerization (CRP) applications in synthesizing degradable sulfur-rich polymers.

### Radical copolymerization to synthesize sulfur-containing polymers

Radical copolymerization, leveraging its process compatibility and monomer versatility, serves as the core technology for introducing sulfur-containing functional units into traditional polymer backbones. In 2023, Watanabe and Kamigaito developed a metal-free direct radical copolymerization strategy for thioamides (such as AcMeThiFA) (**50**) and vinyl monomers (**49**) [61]. The reaction is initiated by AIBN to enable alternating addition of C=S and C=C bonds, constructing thioether linkages (–CH_2_–S–) in the main chain (Fig. 7b). Key innovations include: (1) LiNTf_2_ salt weakens C=S conjugation through coordination, significantly enhancing thioamide incorporation in styrene systems; (2) highly reactive monomers (e.g. vinyl acetate) tend to form alternating-rich sequences (AcMeThiFA: vinyl acetate = 58:42); (3) thioether bonds undergo selective cleavage in AgNO_3_ solution at room temperature within 24 h, with the degree of degradation positively correlated with thioamide content. In 2022, Kopeč’s group prepared fully degradable crosslinked polyacrylate networks via conventional radical copolymerization in an n-butyl acrylate/anisole system (Fig. 7c) [62]. This was achieved by incorporating 4 mol% of dibenzo[*c, e*]oxepane-5-thione (DOT) as a backbone-cleaving comonomer and adding 1 mol% of the crosslinker hexanediol diacrylate. Owing to selective cleavage of thioester bonds, the networks underwent complete dissolution after 24 h of aminolysis in a 5.8 M isopropylamine/THF solution. Although DOT significantly retarded polymerization kinetics and reduced the *M*_n_ of primary chains, key physical properties—including mechanical performance, swelling behavior, and thermal stability—remained virtually unaffected. This study is the first to validate the ‘reverse gel-point model’ in a free-radical polymerization system: when the dispersity of degradation fragments is ∼5, a DOT-to-crosslinker molar ratio of ≥4:1 is required to achieve complete degradation.

In 2023, Destarac and Ivanchenko incorporated thionoglycolide (TGD) into the backbones of poly(tert-butyl acrylate) or polystyrene via radical copolymerization, confirming 100% insertion in the cyclic thioketal form to form degradable *S, O*-thioketal linkages (**56**) [63]. These linkages underwent efficient cleavage (>98% degradation) within 20 min using NaOCl/NaOMe (Fig. 7d). Unsubstituted TGD demonstrated optimal copolymerization performance (*M*_n​_ up to 111 kDa), offering a new strategy for designing highly reactive and rapidly degradable vinyl plastics. In 2024, Destarac and colleagues utilized the commercial reagent 1,1′-thiocarbonyldiimidazole (TCDI) to successfully synthesize degradable vinyl polymers with diimidazolyl thioether units in the main chain via radical copolymerization (Fig. 7e) [64]. This work for the first time achieved efficient random copolymerization of TCDI with monomers such as tert-butyl acrylate (*t*BA) and styrene, with TCDI incorporation rates of 12%–14%. The resulting polymers could undergo partial degradation when treated with bleach, amines, or bases (with the highest *M*_n_ reduction reaching 68%), attributed to the multi-mechanistic cleavage of thioether/thioester bonds. This provides a new strategy for simplifying the design of degradable polyolefins.

Despite the advantages of radical copolymerization, such as simple operation, direct introduction of degradable thioether bonds into the polymer backbone, and endowing materials with tunable thermal properties (e.g. increased *T*_g_) and degradability, it still faces significant challenges: large differences in reactivity between monomers (e.g. low incorporation ratio of thioamides during copolymerization with stable vinyl radicals), possible *β*-fragmentation side reactions, degradation relying on non-environmental conditions such as heavy metal salts, difficulty in balancing thermal stability and degradability, insufficient characterization of material mechanical properties, and cost and process complexity in large-scale production.

### Reversible addition-fragmentation chain transfer (RAFT) synthesis of sulfur-containing polymers

Compared to the uncontrolled chain growth process of traditional free radical polymerization, synthesized sulfur-containing polymers often exhibit limitations such as random backbone structures, broad molecular weight distributions (*Đ* >1.5), and missing functional sites. RAFT polymerization [65,66], as a representative technique of controlled/living CRP, revolutionarily overcomes the control precision limitations of traditional methods by introducing efficient chain transfer agents (CTAs). This provides a powerful tool for synthesizing degradable sulfur-containing polymers with highly defined structures and programmable degradation behavior. The core of the RAFT process lies in its unique reversible chain transfer mechanism. This mechanism achieves precise control over polymer molecular weight, molecular weight distribution, and topology, while maintaining the advantage of broad monomer adaptability inherent in free radical polymerization. Crucially, RAFT technology exhibits exceptional tolerance for sulfur-containing functional groups (such as thioethers, thioesters, and even disulfide bonds serving as degradation triggers), making it particularly suitable for constructing sulfur-containing polymer systems.

RAFT polymerization revolutionized the design of branched structures through precise positioning of dynamic covalent thioester bonds. In 2022, Becker’s group synthesized star-shaped poly(methyl methacrylate) (PMMA) via the RAFT arm-first approach using a bifunctional thioester crosslinker, achieving thioester bond-mediated controllable degradation for the first time (Fig. 7f) [67]. These polymers (**61**) completely degraded into linear PMMA arms via amidation under mild conditions (150°C, 2 h, 50 equivalents hexylamine), exhibiting a degradation rate 100 times faster than traditional ester-bond crosslinked counterparts. Their highly branched architecture (low viscosity factor *g’* down to 0.22) and thermal stability (5% weight loss temperature ∼300°C) provide novel strategies for biomedical carriers (e.g. controlled drug release) and recyclable material design.

In 2025, Tan’s group developed segmented hyperbranched polymers with chain-extendable branching points via wavelength-selective photo-controlled RAFT polymerization [68]: using a 530 nm light source to activate the RAFT copolymerization of a trithiocarbonate-derived dimethacrylate (CDMA) with poly(ethylene glycol) methyl ether methacrylate (PEGMA), achieving precise control over the branching degree (when [PEGMA]/[CDMA]/[chain transfer agent] = 25/4/1, *M*_n_ = 13.9 kDa, *Đ* = 2.26). Switching to blue light (465 nm) activated the trithiocarbonate groups at the branching points, enabling simultaneous chain extension with DMA (*M*_n_ increased to 14.4 kDa). These polymers could be completely degraded into linear chains (*Đ* reduced to 1.21) upon treatment with hydrazine for 3 h. Furthermore, they served as macromolecular RAFT agents to mediate the polymerization-induced self-assembly (PISA) of styrene, forming nanoparticles ranging from spheres to porous vesicles. This approach overcame the flocculation bottleneck associated with traditional crosslinkers, offering a new strategy for degradable nanodrug carriers.

In 2025, Roth’s group achieved dual precise control of polymer degradation rates and phase transition temperatures by designing substituents on seven-membered cyclic allylic sulfide lactones (Fig. 7g) [69]. Utilizing RAFT RROP with methacrylamides (HPMAm/NIPMAm) and oligo(ethylene glycol) methyl ether methacrylate (OEGMA)—employing the trithiocarbonate 4-cyano-4-[(dodecylsulfanylthiocarbonyl)sulfanyl]pentanoic acid as the chain transfer agent and AIBN as the initiator—a series of copolymers were prepared. The benzannulated monomer (**63**) introduced backbone phenyl esters that underwent selective aminolysis degradation with isopropylamine, while aliphatic ester linkages remained stable. Simultaneously, the hydrophobicity of the substituents significantly lowered the lower critical solution temperature of OEGMA copolymers. The furanyl group retained potential for post-polymerization modification. This strategy provides a material platform for controlled drug release and tissue engineering, enabling independent customization of degradation kinetics and stimulus responsiveness.

In summary, RAFT polymerization has become a core tool for synthesizing sulfur-containing polymers by virtue of its precise molecular weight control, high functional group tolerance, and retention of active chain ends. This has successfully enabled innovative systems such as controlled sulfur radical conversion, thioester crosslinking/degradation, and main-chain thioether incorporation. However, this technology still faces drawbacks including monomer limitations, degradation difficulties, and insufficient biocompatibility. Future efforts require the integration of intelligent sulfur chemistry and dynamic topological engineering: designing strained thiolactones to enhance reactivity, developing photo/enzyme-responsive degradation switches to achieve precise cleavage in physiological environments, and coupling the redox responsiveness of disulfide bonds to construct recyclable medical materials. This will propel degradable polyolefins from ‘controlled synthesis’ towards a ‘biologically intelligent closed-loop’.

### Multifunctional applications

Radical polymerization, particularly the RAFT technique, provides a powerful tool for preparing structurally well-defined sulfur-containing functional polymers. By introducing backbone-degrading units (e.g. DOT) or utilizing the RAFT agent itself, fully degradable acrylate networks and precisely controlled block copolymers can be prepared, offering new paradigms for the design of recyclable adhesives and materials with tailored degradation [62,68]. More importantly, the advantage of RAFT in controlling polymer self-assembly enables the preparation of nanoparticles with precise size and morphology, which hold unique value as biodegradable nanocarriers for drug delivery [69]. Additionally, the incorporation of thioether linkages via copolymerization lays the foundation for preparing stimuli-responsive coatings and functional elastomers [63,70].

## INNOVATIVE POLYMERIZATION METHODOLOGIES INVOLVING DIRECT SULFUR PARTICIPATION

### Innovative polymerization methodologies involving direct S_8_

**Figure 8.**
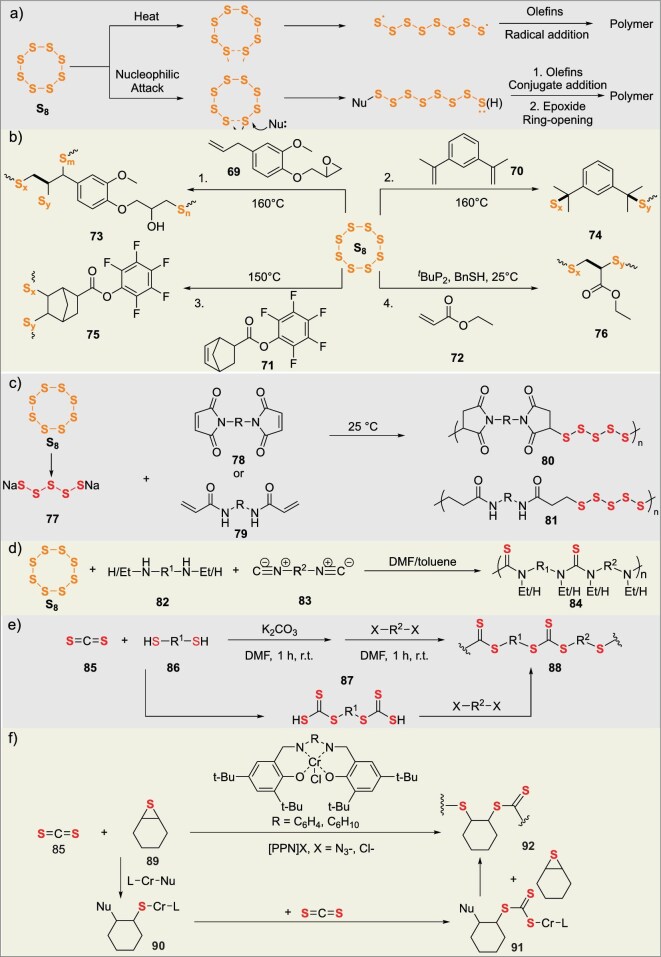
(a) Mechanisms of sulfur participation in sulfur-containing polymer synthesis. (b) Sulfurization and crosslinking of olefins mediated by S_8_. (c) Sulfurization polymerization of S_8_ with alkenyl-containing heterocycles/amides. (d) Multicomponent sulfurization polymerization of S_8_, diamines, and diynes. (e) Sulfurization condensation/polymerization of S=C=S with thiols. (f) Coordination polymerization and sulfurization cycling of S=C=S with thiacycles.

Elemental sulfur is inexpensive and readily available as a byproduct of coal chemical processes. Its utilization in modifying rubber or synthesizing sulfur-containing polymers enables high-value conversion of elemental sulfur. Elemental sulfur primarily exists in the S_8_ form, which readily undergoes ring-opening at high temperatures to generate highly reactive diradical sulfur species or is attacked by nucleophiles to produce sulfur anions or thiyl groups. This reactivity enables two direct strategies for S_8_ polymerization: inverse vulcanization [10] and anionic hybrid copolymerization. By varying the structure of the alkene monomer and the ratio of sulfur atoms to alkene monomers, these methods yield a diverse range of complex sulfur-containing polymers (Fig. 8a).

In 2025, Tan’s group achieved catalyst-free copolymerization of S_8_ with bio-based eugenol glycidyl ether (EGE) and 10-undecenol glycidyl ether (UGE), forming dynamically cross-linked epoxy resin networks (SxEyUz) (**73**) (Fig. 8b-1) [68]. The resulting materials exhibit a tensile strength of 13.0 MPa, tunable *T*_g_ of 12–38°C, and thermal decomposition at ∼200°C, making them suitable for recyclable carbon fiber-reinforced composites. During polymerization, the allylic protons of EGE generate thiol groups, triggering ring-opening and cross-linking of epoxy groups to form post-curable intermediates. The SxEyUz networks degrade in methanol solutions of Na_2_S or neat hexylamine, enabling closed-loop recycling of carbon fiber composites.

In 2023, Pyun’s group copolymerized S_8_ with 1,3-diisopropenylbenzene (DIB) to form a high sulfur content (50 wt%) polysulfide copolymer (poly(S-r-DIB)) (**74**) [71]. The obtained material has a *T*_g_ of ∼25°C and a refractive index >1.75, making it suitable for applications in infrared optical materials, lithium-sulfur battery electrodes, self-healing materials, and other fields. Through solid-state/liquid-state NMR, synthesis of model compounds, and DFT calculations, the understanding of the conventional polymerization mechanism was revised. It was demonstrated that the polymerization mechanism involves sulfur radical-mediated allylic hydrogen abstraction from DIB, forming thiocumyl units, rather than the initially proposed bis-sulfurated structure. The poly(S-r-DIB) can be reductively degraded by LiAlH_4_ to release thiocumyl fragments, verifying the dynamic sulfur bond characteristics (Fig. 8b-2). Théato’s group copolymerized S_8_ with norbornenyl pentafluorophenyl ester (NB-PFPE) (**71**) to synthesize a high-sulfur-content polymer (poly(S-r-NB-PFPE)) (**75**) [72]. This polymer enables the introduction of diverse functional groups—such as polyethylene glycol (PEG), silanes, and allylamine—via amide-based post-polymerization modification (PPM) of the active ester, thereby tailoring material properties. The resulting cross-linked network materials feature tunable *T*_g_ and are applicable as: aqueous nanoparticles, hydrophobic coatings, mercury adsorbents. Degradation of the poly(S-r-NB-PFPE) may occur through dynamic cleavage of S–S bonds at elevated temperatures (Fig. 8b-3). Zhang’s group copolymerized S_8_ with acrylates (such as ethyl acrylate EA or 1,4-butanediol diacrylate BDDA) at 25°C via anionic hybrid copolymerization to form polymers containing short polysulfide segments (**76**) (1–4 sulfur atoms) [73]. The polymerization mechanism involves dynamic chain-transfer reactions between sulfur anions and carbon anions, avoiding the formation of long polysulfide segments. The resulting cross-linked polymer (P(BDDA_33_-S_77_)) exhibits a tensile strength of 10.7 MPa and a breaking strain of 22%, and can achieve room-temperature self-healing and cold-pressing reprocessing via dynamic sulfur bonds, making it suitable for high-performance elastomers, self-healing materials, and other fields (Fig. 8b-4).

Regardless of whether the mechanism is ionic or radical, the high similarity between S_X_ species and S–S bonds in polymer chains makes the addition of S_8_ to olefins inevitably result in uncertain polythiol chain lengths, affecting polymer properties. In 2024, Erdemi’s group generated polyamide and polyimide copolymers with polythiol chains in the main chain through stepwise polymerization of Na_2_S_5_, bisacrylamide (**79**), or bismaleimide (**78**) monomers under ambient temperature and catalyst-free conditions [74]. The obtained polymers have a molecular weight of up to 31.8 kDa and a monomer conversion rate of up to 94%, with adjustable thermal stability and *T*_g_. Crosslinked network materials can be further prepared using a crosslinker, with storage moduli (*G*′) reaching 10^2^–10^4^ Pa and exhibiting 37%–48% Hg^2+^ adsorption rates in water (Fig. 8c). Tang’s group developed a method for the one-step conversion of S_8_, aliphatic diamines, and diisocyanides into functional polythioureas (**84**) at room temperature through catalyst-free multicomponent polymerization [75]. This polymerization features high efficiency, high atom economy, and mild reaction conditions, and can be conducted in air or nitrogen. The resulting polymers exhibit molecular weights up to 242 500 g mol^−1^ and yield up to 95%. The polythioureas decompose at 209–279°C, dissolve in polar solvents such as *N,N*-Dimethylformamide (DMF) and dimethyl sulfoxide (DMSO), and can be used for highly sensitive detection and efficient removal of mercury ions, with real-time monitoring of mercury contaminant removal through fluorescence changes (Fig. 8d).

### Innovative polymerization methodologies involving direct CS_2_

Precise control over the introduction of sulfur atoms in polymers can be achieved using carbon disulfide (CS_2_) as a sulfur source to introduce dithiocarbonate fragments. In 2025, Tang’s group efficiently synthesized polytrithiocarbonates (PTTCs) (**88**) via multicomponent tandem polymerization at room temperature using CS_2_, dithiols, and alkyl dihalides as monomers and K_2_CO_3_ as a catalyst, with a yield of 93% and a *M*_n_ of 37.9 kDa (Fig. 8e) [76]. PTTCs exhibit a thermal decomposition temperature of 288°C, a tensile strength of 87.5 MPa, high crystallinity, unique upper critical solution temperature behavior, and aggregation-induced emission characteristics, making them applicable in elastomers, high-performance plastics, and thermoresponsive fluorescent materials. Losio’s group utilized chromium complexes salphen-CrCl and salen-CrCl to catalyze the copolymerization of cyclohexene sulfide (**89**) and CS_2_, efficiently synthesizing sulfur-rich poly(trithiocyclohexylcarbonate) (**90**) (PCS) (Fig. 8f) [77]. The polymerization was conducted at room temperature or 50°C using [PPN]^+^X^−^ salts as cocatalysts, with a maximum yield of 80% and a molecular weight of up to 18 kDa. PCS decomposes at ∼250°C, has a *T*_g_ of 80°C, a refractive index >1.72, and exhibits effective antibacterial activity against *Escherichia coli* and *Staphylococcus aureus*.

### Multifunctional applications

Polymerization directly utilizing inexpensive sulfur sources like S_8_ and CS_2_ is a shortcut to achieving high-value transformation of sulfur and producing high-performance materials. High-sulfur-content polymers prepared via strategies such as inverse vulcanization not only possess a high refractive index and good infrared transmittance, making them suitable for infrared optical lenses and coatings, but can also serve as cathode materials for lithium-sulfur batteries [72]. Furthermore, polymers constructed with polysulfide chains (Na_2_S_5_) or CS_2_ exhibit efficient chelating ability towards heavy metal ions like Hg^2+^ due to the sulfur atoms in their backbone, giving them broad application prospects as adsorbents for water treatment [75,76]. Sulfur-containing polymers synthesized by these methods often possess dynamic properties, enabling their use in recyclable composite materials and self-healing sealants [72,74].

## SYNTHESIS OF SULFUR-CONTAINING POLYMERS MEDIATED BY CLICK CHEMISTRY

### Recyclable poly(dithioacetal)s via acetal chemistry

**Figure 9.**
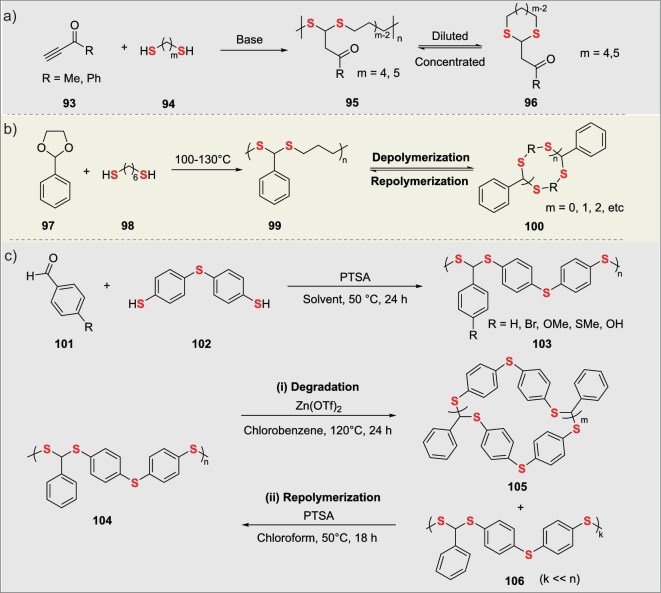
(a) Click-type sulfurization polymerization of alkynes with dithiols. (b) Click-based dynamically reversible sulfurization polymerization of epoxides with dithiols. (c) Click-inspired sulfurization polycondensation and recycling conversion of aldehydes with dithiols.

Leveraging the reversibility of dithioacetals, polymer materials with both high performance and degradability can be obtained. In 2021, Li’s group dynamically polymerized 3-butyn-2-one (**93**) or 1-phenyl-2-propyn-1-one with 1,4-butanedithiol (**94**) via base-catalyzed thiol-yne ‘click’ copolymerization at high concentrations to generate poly(dithioacetals) (**95**) [78]. The poly(dithioacetals) can degrade into seven-membered cyclic dithioacetals via retro-Michael addition in dilute alkaline solutions and repolymerize at high concentrations. The poly(dithioacetals) exhibit decomposition temperatures of 188–217°C and *T*_g_ of −47 to −16°C, suitable for the design of recyclable materials (Fig. 9a). Oyaizu’s group prepared an aromatic poly(dithioacetal) (PDTA) through protonic acid-catalyzed polycondensation of aldehydes (**97**) and dithiols (**98**) [79]. PDTA features a refractive index >1.7, a *T*_g_ >60°C, and visible light transmittance ≥95%. It can be selectively degraded into low-molecular-weight cyclic products catalyzed by the Lewis acid Zn(OTf)_2_ and repolymerized via protonic acid catalysis for closed-loop recycling, with nearly unchanged optical properties after recycling, making it suitable for encapsulating materials in high-performance and degradable eco-friendly optoelectronic devices (Fig. 9b).

In 2024, Ma’s group synthesized poly(dithioacetals) (**103**) via a catalyst-free, solvent-free acetal-thiol click reaction at ∼80°C [80]. The polymerization leveraged dynamic covalent acetal-thiol exchange, yielding linear polymers with *M*_w_ up to 110 kDa. Semi-interpenetrating polymer networks (SIPNs) were prepared by thermal processing or reactive extrusion. Degradation exploited dynamic bond reversibility, enabling depolymerization into cyclic dithioacetals upon heating and subsequent closed-loop repolymerization (**106**). The SIPNs exhibited a tensile strength of 28 MPa (comparable to engineering thermoplastics) and *T*_g_ ≈ −25°C, showing promise for sustainable materials, self-healing networks, and biomedical applications (Fig. 9c).

### Functional polymers via multifunctional thiol-mediated click reactions

**Figure 10.**
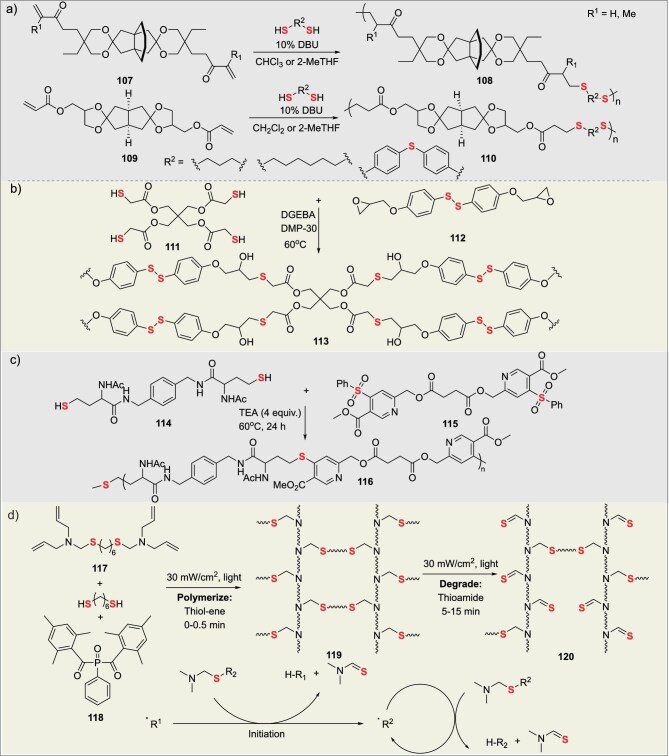
(a) Synthetic approaches based on thiol-mediated conjugate addition, (b) epoxide ring-opening, (c) S_N_Ar aromatic nucleophilic substitution, and (d) photo-controlled polymerization and photo-controlled degradation.

Thiosulfhydryl exhibits high nucleophilicity, capable of undergoing conjugate addition, epoxy ring-opening, and S_N_Ar (nucleophilic aromatic substitution) reactions. In 2022, Vares’s group converted rigid spirocyclic diols derived from citric acid into di(meth)acrylates via thiol-Michael addition ‘click’ reactions, and copolymerized them with 1,6-hexanedithiol, 1,3-propanedithiol, or aromatic dithiols to prepare poly(*β*-thioether ester ketals) (**107**) [81]. The resulting polymers (**109**) have decomposition temperatures above 300°C and *T*_g_ ranging from −7 to 40°C. They can be efficiently depolymerized into the original diketones and thiol monomers via acid-catalyzed hydrolysis of ketal bonds in a 1 M HCl/acetone mixture, enabling chemical recycling (Fig. 10a). Chen’s group prepared recyclable polymer-bonded explosives through mild ring-opening reactions of disulfide-based epoxy monomers with thiol curing agents, ensuring compatibility with the high-energy explosive HMX [82]. The polymer exhibits a compressive strength of 35.1 MPa and excellent thermal stability. It can be non-destructively degraded via thiol-disulfide exchange reactions in neutral thiol solutions (dithiothreitol/CH_2_Cl_2_) at room temperature to recover HMX (Fig. 10b).

In 2025, Liu’s group synthesized pyridinyl polythioethers (**116**) via one-pot thiolactone ring-opening of *N*-acetyl homocysteine thiolactone (NHTL) with diamines to generate dithiol monomers (**114**), followed by click step-growth polymerization with bis(phenylsulfone) (**115**) monomers [83]. The polymer features thermal stability and clustering-triggered emission (CTE), and can be oxidized by H_2_O_2_/Na_2_WO_4_ to convert thioether groups into sulfoxide/sulfone moieties, followed by ‘declick’ reactions with 2-mercaptoethanol for controlled degradation. It can be applied as a fluorescent probe for Cu^2+^ detection (Fig. 10c). Bowman’s group developed a novel light-responsive polymer system based on photo-cleavable thioaminals, achieving ‘polymerization-degradation’ two-stage materials regulated by a single light source through combining thiol-ene click polymerization with radical-mediated thioaminals scission [84]. Polymerization employs thiol-ene photopolymerization (400–500 nm illumination) to rapidly form crosslinked networks (**119**) within 0.5 min. Degradation triggers *β*-scission of thioaminals to generate thioamides and break polymer C–S bonds via continuous irradiation with the same light source for 5–15 min. This polymer exhibits lithographic compatibility and can serve as positive/negative photoresists for 3D printing and micropatterning (Fig. 10d).

### Dynamic networks via thiol-isocyanate click chemistry

**Figure 11.**
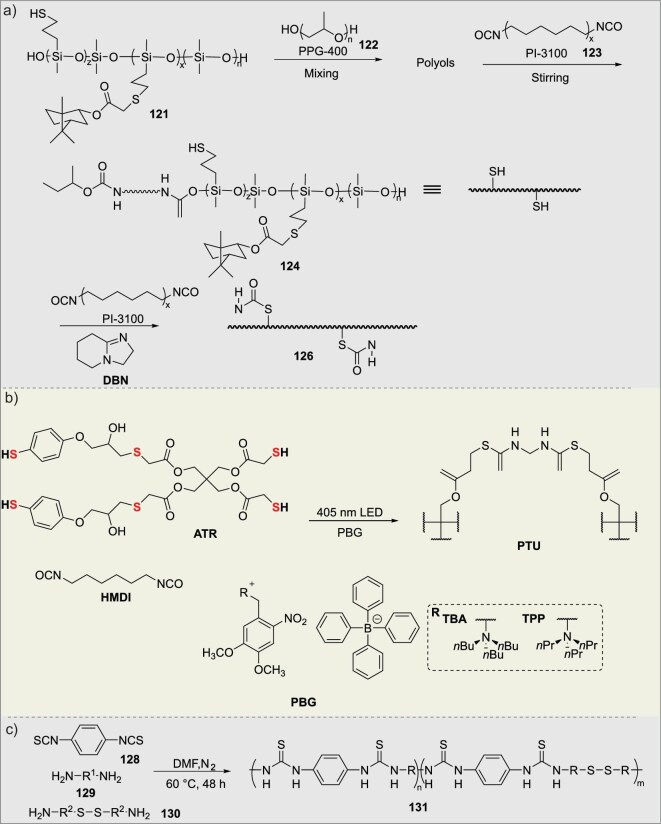
(a) Dynamic covalent crosslinking and network construction of sulfur-containing siloxanes with polyisocyanates. (b) Photo-triggered dynamic covalent polymerization and network regulation of thiol-containing compounds. (c) Dynamic covalent polymerization and network formation from diisothiocyanates and disulfide-containing diamines.

Dynamic networks constructed via thiol-isocyanate click chemistry integrate the efficiency and selectivity of the reaction with the unique characteristics of the resulting dynamic covalent thiourethane bonds, offering a powerful and versatile platform for developing a new generation of reprocessable and recyclable polymeric materials. In 2023, Liu’s group developed a series of terpene polysiloxane-based poly(thiourethane-urethane) materials via thiol-ene and thiol-isocyanate click polymerizations [85]. The incorporated dynamic thiourethane bonds, with an activation energy of 125 kJ mol^−1^, enabled excellent self-healing (scratch recovery at 90°C) and reprocessability (Fig. 11a). Leveraging the synergistic effect of the rigid isobornyl acrylate ring and hydrophobic polysiloxane, the optimized poly(thiourethane-urethane) (**126**) exhibited significantly enhanced hydrophobicity (water contact angle increased to 102°), reduced water absorption (8.3%), and a remarkable tensile strength of 12.56 MPa. This work presents a sustainable strategy for creating high-performance, healable, and recyclable polymer coatings, demonstrating the potential of dynamic covalent chemistry and bio-based hybrids in designing durable functional materials.

In 2024, Page’s group developed a visible-light-activated polymerization system based on onium photocages [86]. By triggering the rapid release of organophosphine catalysts under solvent-free, 405 nm light conditions, they achieved high-efficiency, spatiotemporally controlled step-growth polymerization of thiol-isocyanate (maximum rate 1280 mM s^−1^) (Fig. 11b). This technology was successfully applied to digital light processing (DLP) 3D printing, enabling the rapid fabrication of PTU objects with an extreme range of properties—from high-strength (*σ*_max_ = 54 MPa, *E_y_* = 1.5 GPa) rigid structures to highly elastic (*ε_b_* = 200%, *E_y_* = 1 MPa) bodies with >99% elastic recovery—using exposure times of just seconds per layer. This provides an innovative material solution for high-performance, high-precision photocuring manufacturing that surpasses traditional acrylates. In the same year, Liu’s group developed a series of polythiourea-disulfide (PTUS*_X_*) (**131**) elastomers via amine-isothiocyanate click polymerization [87]. The material ingeniously integrates the synergistic exchange of three types of dynamic bonds—disulfide, thiourea, and hydrogen bonds—enabling efficient self-healing at room temperature (achieving 96% healing efficiency after 48 h) (Fig. 11c). The optimized PTUS_8_ (**128**:**129**:**130** = 4:3:1) sample exhibits remarkable mechanical properties (tensile strength of 12.9 MPa) and flexibility, with an amorphous structure that facilitates chain mobility. Furthermore, by combining PTUS_8_ with lithium salts, the team fabricated a self-healing solid polymer electrolyte with an ionic conductivity of up to 8.4 × 10^−4^ S/cm at 100°C, which could recover its conductivity after damage. This work demonstrates the potential of PTUS_8_ for applications in safe and durable solid-state lithium batteries, offering a novel strategy for designing high-performance self-healing polymers through multi-dynamic bond synergy.

### Multifunctional applications

Synthesis strategies based on click chemistry are known for their efficiency and modularity, providing convenience for the rapid construction of functionalized sulfur-containing polymers. Polymers obtained through the polymerization of dynamic dithioacetals combine high refractive index, high transmittance, and chemical recyclability, making them ideal candidates for encapsulation materials in next-generation green optoelectronic devices [80,81,85]. Click reactions like thiol-ene/thiol-epoxy can rapidly build crosslinked networks, which, when used as degradable polymer-bonded explosives and temporary protective coatings, achieve a unity of functionality and environmental friendliness. More interestingly, click polymerization allows for the precise incorporation of groups like pyridine into the polythioether backbone, enabling the development of smart materials for chemical sensing and fluorescent detection [83].

## DYNAMIC COVALENT POLYMERIZATION

### Synthesis and degradation of dynamic covalent networks

**Figure 12.**
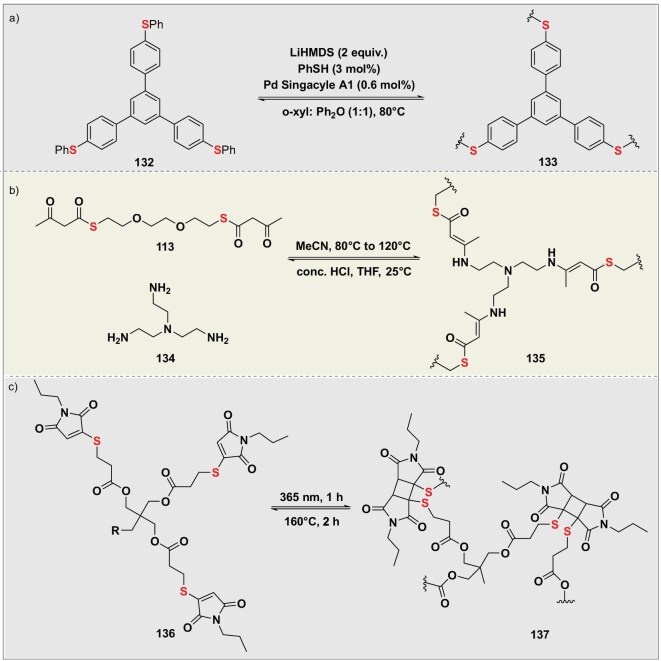
(a) Pd-catalyzed dynamic covalent exchange of sulfur linkages and network rearrangement in aryl thioethers. (b) Dynamic sulfur-based crosslinking and network reconfiguration of thioesters with polyamines. (c) Light/heat dual-triggered polymerization and network reorganization via sulfur-containing heterocycle dynamic covalent bonds.

Over recent years, significant strides have been made in developing closed-loop recyclable polymers with robust performance [88]. Pioneering work utilizing dynamic covalent chemistry has demonstrated the feasibility of creating high-performance materials that can be efficiently recovered and reprocessed without sacrificing stability or functionality. In 2021, Morandi’s group successfully prepared porous poly(aryl thioether) materials (**122**) integrating high stability and closed-loop recyclability under mild conditions using a reversible palladium-catalyzed C–S/C–S metathesis reaction [89]. These polymers formed through dynamic covalent bond exchange and could be efficiently depolymerized via cyclohexanethiol mediation to recover the original monomers. The materials exhibited polyphenylene sulfide (PPS)-grade stability, withstanding 450°C high temperatures and corrosion by strong acids/oxidants, while featuring a tunable specific surface area of 192 m^2^ g^−1^. The high-density thioether linkages within their framework endowed them with three key functions: exceptional noble metal ion capture capability, achieving adsorption capacities of 94 mg g^−1^ for Pd^2+^ and 147 mg g^−1^ for Au^3+^; ultrasensitive Pd^2+^ detection at the 2 ppb level via fluorescent variants; and efficient heterogeneous catalysis when loaded with palladium, maintaining unchanged activity over six cycles in alkyne semi-hydrogenation reactions (Fig. 12a).

In 2023, Zhou’s group developed closed-loop recyclable covalent adaptable networks (CANs) (**124**) using vinylogous carbamothioate (VC) bonds [90]. Synthesized via polycondensation, these CANs achieve a high refractive index (1.600) and rapid stress relaxation (120.6 s at 100°C). Room-temperature acidolysis (12 M HCl) depolymerizes networks, recovering pure monomers (81% yield) and cross-linkers (86% yield). Remanufactured CANs retain original performance. A key insight of this approach is that segmental relaxation dominates dynamics despite slower intrinsic VC exchange kinetics (activation energy: 46.1 kJ mol^−1^), enabling efficient network reconfiguration (Fig. 12b).

In 2024, Houck’s group developed an intrinsically recyclable bulk covalent network material (**137**) based on the orthogonal photo-thermal reversibility of monothiomaleimides [91]. The material undergoes UV-triggered [2 + 2] photodimerization crosslinking and quantitatively reverts to monomers via thermal cycloreversion above 120°C. Both solution and bulk systems achieved over five efficient cycles with >98% conversion, exhibiting a storage modulus of 0.8 MPa after photocuring and retaining >95% mechanical performance after multiple recycling rounds. This research overcomes the longstanding challenge of achieving closed-loop recycling for traditional photoreversible materials in bulk state, providing a groundbreaking solution for designing high-performance recyclable thermosets (Fig. 12c).

### Multifunctional applications

Dynamic covalent chemistry enables development of advanced materials combining robust performance with complete recyclability. Porous poly(aryl thioether)s fabricated through reversible C–S/S–S exchange demonstrate exceptional performance in precious metal recovery and heterogeneous catalysis applications, enabling direct transformation of waste streams into functional catalysts [87]. Covalent adaptable networks based on vinylogous carbamothioate bonds or photodimerizable thiomaleimides address the critical challenge of thermoset recyclability, providing transformative solutions for high-performance composites, coatings, and 3D-printed resins with full chemical recyclability [90,91]. The diverse synthetic strategies and controllable degradation mechanisms outlined above pave the way for various practical applications. The performance of these sulfur-containing polymers in specific fields, quantified by key metrics, is summarized for representative polymers in Table 5.

**Table 5.**
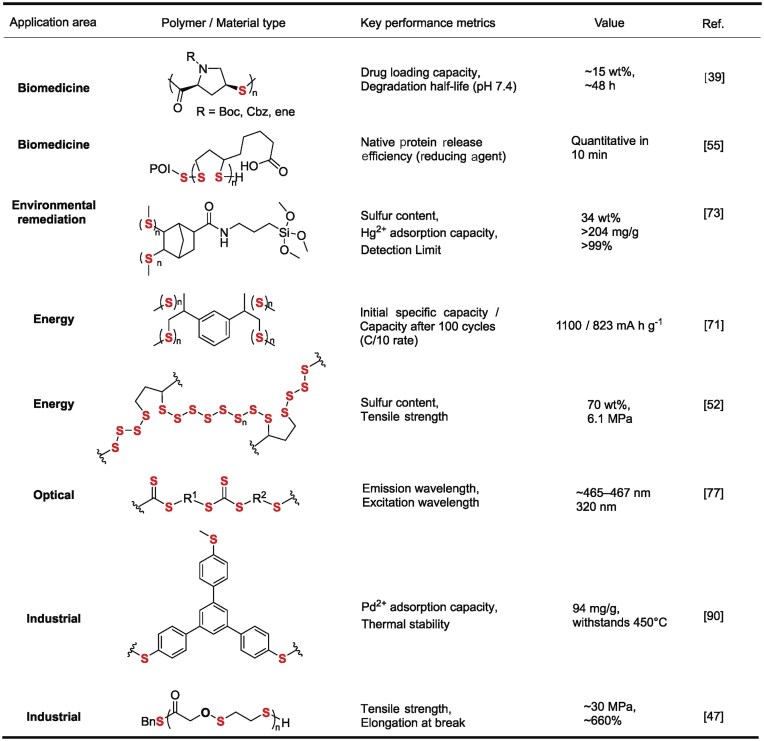
Performance metrics of sulfur-containing polymers in multifunctional applications.

### Summary

In summary, dynamic covalent chemistry enables the creation of closed-loop recyclable polymers with industrial-grade stability, multifunctionality, and monomer recovery under mild conditions. However, limitations include dependency on noble metal catalysts, sensitivity to processing constraints, and challenges in scalability. Future improvements require developing catalyst-free or earth-abundant catalyst systems, designing bonds with enhanced environmental stability, optimizing trigger efficiency, and establishing robust bulk reprocessing techniques. Overcoming these barriers will unlock the full potential of dynamic covalent networks for sustainable high-performance materials.

## CONCLUSIONS AND OUTLOOK

**Figure 13.**
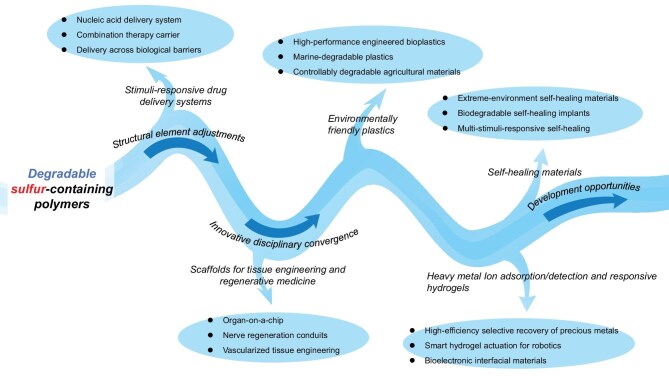
Development and prospect of sustainable sulfur-containing polymers.

This review systematically summarizes the latest advances in the synthesis strategies, degradation mechanisms, and multifunctional applications of sulfur-containing sustainable polymers. Through precision synthesis techniques such as ROP and RAFT polymerization, dynamic covalent bonds such as thioethers and thioesters have been successfully incorporated into polymer backbones, endowing the materials with unique functionalities such as controllable degradability, closed-loop recyclability, and high refractive indices. Leveraging these properties, sustainable sulfur-containing polymers demonstrate broad application prospects across multiple fields (Fig. 13): in biomedicine [92], they serve as smart drug carriers, degradable tissue engineering scaffolds, and gene delivery systems, enabling targeted drug release and biocompatibility; in environmental protection, they are used for degradable packaging, agricultural mulch films, and heavy metal adsorption, promoting plastic closed-loop recycling; in optical and electronic devices [93], high-refractive-index materials enhance optical performance, and dynamic covalent network materials enable self-healing; in the energy sector, they are applied in solid-state lithium battery electrolytes and sulfur-based cathode materials; in industry [94], they are used as anti-scaling agents and flame retardants. In the future, with breakthroughs in low-cost monomer synthesis, stereochemical control, and full–life-cycle recycling technologies, they will play a more significant role in cutting-edge fields such as biomimetic synthesis and space environmental protection, becoming a core direction for ‘green polymers’.

Despite significant progress, several core challenges and opportunities must be addressed to translate sustainable sulfur-containing polymers from the laboratory to practical applications and ultimately realize their environmental and economic benefits:

1. Core challenge: balancing durability during use with controllable degradability at end-of-life.

This is an inherent ‘performance paradox’ in the field of degradable materials. Future research should focus on resolving this contradiction through precise molecular design. For example, dynamic sulfur-based bonds with ‘switch-like’ responsiveness to specific external stimuli (e.g. specific pH, enzymes, or light wavelengths) can be designed. These bonds would remain highly stable under normal conditions of use, ensuring material durability, yet be rapidly and selectively activated in specific waste treatment environments to trigger efficient degradation. Developing theoretical models to predict the relationship between polymer durability and degradability will be a key tool guiding molecular design.

2. Frontier opportunity: integrating sulfur chemistry with bio-based monomers and circular design.

To achieve true sustainability, focusing solely on ‘end-of-pipe degradation’ is insufficient; efforts must start from the source. An important direction is the development of sulfur-containing monomers derived from biomass (e.g. lignin derivatives, amino acids, fatty acids). This not only reduces reliance on fossil resources but also leverages the chiral centers present in biomass to achieve stereoregular polymerization, enhancing material properties. Simultaneously, ‘upcycling’ strategies should be actively explored. For instance, polymer degradation products (e.g. thiols) could be directly converted into higher-value chemicals (e.g. pharmaceutical intermediates or functional small molecules) rather than simply being recycled into monomers, thereby enhancing the economic viability and resource efficiency throughout the entire life cycle.

3. Practical constraints: overcoming real-world bottlenecks in closed-loop recycling.

Currently, efficient chemical recycling is mostly achieved under ideal laboratory conditions. Scaling up to industrial applications faces severe challenges:

Monomer purity and catalyst residues: in real-world recycling streams, additives, fillers, contamination from other polymers, and catalyst residues can severely impact depolymerization efficiency and the purity of recovered monomers, hindering repolymerization. Developing highly selective catalysts tolerant to impurities, and establishing efficient, low-energy purification processes (e.g. membrane separation, crystallization) are crucial.

Economic feasibility: the synthesis routes for many sulfur-containing monomers are currently complex and costly. Future research should prioritize the development of low-cost monomer synthesis pathways starting from industrial by-products (like elemental sulfur, S₈) or bulk chemicals (like CS₂). Furthermore, comprehensive techno-economic analysis and life cycle assessment of the entire closed-loop process are necessary to demonstrate its long-term competitive advantage over the traditional ‘take-make-dispose’ linear model or mechanical recycling.

Looking ahead, the development of sulfur-containing degradable polymers will transcend the singular goal of ‘replacing conventional plastics’ and move towards the construction of ‘intelligent’, ‘multifunctional’, and ‘circular’ material systems. Through interdisciplinary collaboration, integrating polymer synthesis chemistry with materials science, process engineering, and artificial intelligence, holds the promise of designing a new generation of smart green materials. These materials will not only degrade on command but also sense their environment, perform functions during their lifetime, and ultimately return gracefully into the cycle. They are poised to play an indispensable role in cutting-edge fields such as biomimetic synthesis, soft robotics, and space environmental protection, ultimately guiding polymer materials science into a truly sustainable new era.

## References

[1] Yu Q, Bai L, Jiang X. Disulfide click reaction for stapling of *S*-terminal peptides. Angew Chem Int Ed 2023; 62: e202314379. 10.1002/anie.202314379 37950389

[2] Yu Q, Zhang X, Jiang X. Bilateral unsymmetrical disulfurating reagent design for polysulfide construction. Angew Chem Int Ed 2024; 63: e202408158. 10.1002/anie.202408158 38923731

[3] Harrak Y, Casula G, Basset J et al. Synthesis, anti-inflammatory activity, and in vitro antitumor effect of a novel class of cyclooxygenase inhibitors: 4-(Aryloyl)phenyl methyl sulfones. J Med Chem 2010; 53: 6560–71. 10.1021/jm100398z 20804197

[4] Ilardi EA, Vitaku E, Njardarson JT. Data-mining for sulfur and fluorine: an evaluation of pharmaceuticals to reveal opportunities for drug design and discovery. J Med Chem 2013; 57: 2832–42. 10.1021/jm401375q 24102067

[5] Teall M, Oakley P, Harrison T et al. Aryl sulfones: a new class of γ-secretase inhibitors. Bioorg Med Chem Lett 2005; 15: 2685–8. 10.1016/j.bmcl.2004.12.017 15863342

[6] Manjunatha BR, Gallizioli C, Fornacon-Wood C et al. Sequence control in sulphur-containing ring-opening co- and terpolymerisations. Angew Chem Int Ed 2025; 64: e202507243. 10.1002/anie.202507243 PMC12184296 40323177

[7] Mutlu H, Ceper EB, Li X et al. Sulfur chemistry in polymer and materials science. Macromol Rapid Commun 2019; 40: 1800650. 10.1002/marc.201800650 30468540

[8] Bai L, Jiang X. Smelless/stable/sustainable sulfur chemistry. CCS Chem 2025; 7: 1889–902. 10.31635/ccschem.025.202505568

[9] Meyer B . Elemental sulfur. Chem Rev 2002; 76: 367–88. 10.1021/cr60301a003

[10] Chung WJ, Griebel JJ, Kim ET et al. The use of elemental sulfur as an alternative feedstock for polymeric materials. Nat Chem 2013; 5: 518–24. 10.1038/nchem.1624 23695634

[11] Jin Y, Yu C, Denman RJ et al. Recent advances in dynamic covalent chemistry. Chem Soc Rev 2013; 42: 6634. 10.1039/c3cs60044k 23749182

[12] Kolesnichenko IV, Anslyn EV. Practical applications of supramolecular chemistry. Chem Soc Rev 2017; 46: 2385–90. 10.1039/C7CS00078B 28317053

[13] Wang BS, Zhang Q, Wang ZQ et al. Acid-catalyzed disulfide-mediated reversible polymerization for recyclable dynamic covalent materials. Angew Chem Int Ed 2023; 62: e202215329. 10.1002/anie.202215329 36602285

[14] Zhu T, Lei R, Wang B et al. Bio-sourced 4-aryl-1,2-dithiolanes for recyclable poly(disulfide)s with high performance. Angew Chem Int Ed 2025; 64: e202503677. 10.1002/anie.202503677 40230294

[15] Zhao J, Yue T, Ren B et al. Recyclable sulfur-rich polymers with enhanced thermal, mechanical, and optical performance. Macromolecules 2022; 55: 8651–8. 10.1021/acs.macromol.2c01628

[16] Wang Y, Li M, Chen J et al. O-to-S substitution enables dovetailing conflicting cyclizability, polymerizability, and recyclability: dithiolactone vs. dilactone. Angew Chem Int Ed 2021; 60: 22547–53. 10.1002/anie.202109767 34424604

[17] Wang Y, Li M, Wang S et al. *S*-carboxyanhydrides: ultrafast and selective ring-opening polymerizations towards well-defined functionalized polythioesters. Angew Chem Int Ed 2021; 60: 10798–805. 10.1002/anie.202016228 33605001

[18] Wang H, Wei J, Jiang X et al. Novel polymerizable sulfur-containing benzophenones as free-radical photoinitiators for photopolymerization. Macromol Chem Phys 2006; 207: 1080–6. 10.1002/macp.200600124

[19] Sáiz LM, Prolongo MG, Bonache V et al. Self-healing materials based on disulfide bond-containing acrylate networks. Polym Test 2023; 117 10783.

[20] Razquin I, Iregui A, Orduna L et al. Reprogrammable permanent shape memory materials based on reversibly crosslinked Epoxy/PCL blends. Molecules 2020; 25: 1568. 10.3390/molecules25071568 32235334 PMC7180467

[21] Kausar A, Zulfiqar S, Sarwar MI. Recent developments in sulfur-containing polymers. Polym Rev 2014; 54: 185–267. 10.1080/15583724.2013.863209

[22] Du T, Shen B, Dai J et al. Controlled and regioselective ring-opening polymerization for poly(disulfide)s by anion-binding catalysis. J Am Chem Soc 2023; 145: 27788–99. 10.1021/jacs.3c10708 37987648

[23] Marshall CM, Molineux J, Kang K-S et al. Synthesis of polycyclic olefinic monomers from norbornadiene for inverse vulcanization: structural and mechanistic consequences. J Am Chem Soc 2024; 146: 24061–74. 10.1021/jacs.4c08113 39143005

[24] Narmon AS, Jenisch LM, Rey J et al. Monothiolactide, a new monomer for the synthesis of recyclable, alternating ester-thioester polymers. ChemSusChem 2024; 17: e202400134. 10.1002/cssc.202400134 39031793

[25] Niu B, Zhang L, Tan J. Orthogonal atom transfer radical polymerization and reversible addition–fragmentation chain transfer polymerization for controlled polymer architectures. Macromolecules 2024; 57: 9766–78. 10.1021/acs.macromol.4c01856

[26] Xue H, Lin H, Wang X et al. Self-healing and degradable functionalization of EVA hot-melt adhesives based on disulfide bonds and polymer free radical copolymerization. Polymer 2024; 313: 127721. 10.1016/j.polymer.2024.127721

[27] Zhang S, Wang Y, Huang H et al. A strategy for controlling the polymerizations of thiyl radical propagation by RAFT agents. Angew Chem Int Ed 2023; 62: e202308524. 10.1002/anie.202308524 37478164

[28] Huang R, Zhao Y, Li C et al. Photocatalytic upcycling of polysulfones at ambient conditions. Nat Sustain 2025; 8: 818–26. 10.1038/s41893-025-01569-x

[29] Yang S, Du S, Zhu J et al. Closed-loop recyclable polymers: from monomer and polymer design to the polymerization–depolymerization cycle. Chem Soc Rev 2024; 53: 9609–51. 10.1039/D4CS00663A 39177226

[30] Salman MK, Karabay B, Karabay LC et al. Elemental sulfur-based polymeric materials: synthesis and characterization. J Appl Polym Sci 2016; 133: 43655. 10.1002/app.43655

[31] Higashihara T, Ueda M. Recent progress in high refractive index polymers. Macromolecules 2015; 48: 1915–29. 10.1021/ma502569r

[32] Kim DH, Jang W, Choi K et al. One-step vapor-phase synthesis of transparent high refractive index sulfur-containing polymers. Sci Adv 2020; 6: eabb5320. 10.1126/sciadv.abb5320 32923596 PMC7455493

[33] Crockett MP, Evans AM, Worthington MJH et al. Sulfur-limonene polysulfide: a material synthesized entirely from industrial by-products and its use in removing toxic metals from water and soil. Angew Chem Int Ed 2015; 55: 1714–8. 10.1002/anie.201508708 PMC4755153 26481099

[34] Limjuco LA, Fissaha HT, Kim H et al. Sulfur copolymerization with hydrophilic comonomers as polysulfides in microbeads for highly efficient Hg^2+^ removal from wastewater. ACS Appl Energy Mater 2020; 2: 4677–89. 10.1021/acsapm.0c00725

[35] Liu Y, Ou S, Wu J et al. Continuous flow ring-opening polymerization and ring-opening metathesis polymerization. Eur Polym J 2024; 216**:** 113288. 10.1016/j.eurpolymj.2024.113288

[36] Bannin TJ, Kiesewetter MK. Poly(thioester) by organocatalytic ring-opening polymerization. Macromolecules 2015; 48: 5481–6. 10.1021/acs.macromol.5b01463 27182085 PMC4863702

[37] Mavila S, Worrell BT, Culver HR et al. Dynamic and responsive DNA-like polymers. J Am Chem Soc 2018; 140: 13594–98. 10.1021/jacs.8b09105 30351134

[38] Suzuki M, Makimura K, Matsuoka S-i Thiol-mediated controlled ring-opening polymerization of cysteine-derived *β*-thiolactone and unique features of product polythioester. Biomacromolecules 2016; 17: 1135–41. 10.1021/acs.biomac.5b01748 26845398

[39] Yuan J, Xiong W, Zhou X et al. 4-Hydroxyproline-derived sustainable polythioesters: controlled ring-opening polymerization, complete recyclability, and facile functionalization. J Am Chem Soc 2019; 141: 4928–35. 10.1021/jacs.9b00031 30892027

[40] Shi C, McGraw ML, Li Z-C et al. High-performance pan-tactic polythioesters with intrinsic crystallinity and chemical recyclability. Sci Adv 2020; 6: eabc0495. 10.1126/sciadv.abc0495 32875116 PMC7438104

[41] Wang S, Liu L, Li K et al. Implementing a sulfur-substitution approach toward a high-performance recyclable polythioester. Polym Chem 2025; 16: 987–93. 10.1039/D4PY01425A

[42] Pei W, Liu Y, Yan Q et al. Crystallization/precipitation driven nonequilibrium ring-opening polymerization of thiovalerolactone toward closed-loop recyclable polythioester with excellent barrier properties. Angew Chem Int Ed 2025; 64: e202505104. 10.1002/anie.202505104 40357831

[43] Zhu Y, Li M, Wang Y et al. Performance-advantaged stereoregular recyclable plastics enabled by aluminum-catalytic ring-opening polymerization of dithiolactone. Angew Chem Int Ed 2023; 62: e202302898. 10.1002/anie.202302898 37058315

[44] Wang Y, Zhu Y, Lv W et al. Tough while recyclable plastics enabled by monothiodilactone monomers. J Am Chem Soc 2023; 145: 1877–85. 10.1021/jacs.2c11502 36594572

[45] Stellmach KA, Paul MK, Xu M et al. Modulating polymerization thermodynamics of thiolactones through substituent and heteroatom incorporation. ACS Macro Lett 2022; 11: 895–901. 10.1021/acsmacrolett.2c00319 35786872

[46] Wang M, Ding Z, Shi X et al. Modulating polymerization behaviors of ether–ester monomers and physicochemical properties of poly(ether-alt-ester)s by heteroatom substitutions. Macromolecules 2024; 57: 869–79. 10.1021/acs.macromol.3c02140

[47] Liu L-H, Wang S-Q, Fan H-Z et al. Chemically recyclable poly(thioether-thioester)s via ring-opening polymerization of seven-membered thiolactones. Polym Chem 2025; 16: 972–8. 10.1039/D4PY01442A

[48] Li L-G, Wang Q-Y, Zheng Q-Y et al. Tough and thermally recyclable semiaromatic polyesters by ring-opening polymerization of benzo-thia-caprolactones. Macromolecules 2021; 54: 6745–52. 10.1021/acs.macromol.1c00497

[49] Zhang Q, Deng Y, Luo H et al. Assembling a natural small molecule into a supramolecular network with high structural order and dynamic functions. J Am Chem Soc 2019; 141: 12804–14. 10.1021/jacs.9b05740 31348651 PMC6696886

[50] Deng Y, Huang Z, Feringa B et al. Converting inorganic sulfur into degradable thermoplastics and adhesives by copolymerization with cyclic disulfides. Nat Commun 2024; 15: 3855. 10.1038/s41467-024-48097-4 38719820 PMC11079033

[51] Shi C, Zhang Z, Scoti M et al. Endowing polythioester vitrimer with intrinsic crystallinity and chemical recyclability. ChemSusChem 2023; 16: e202300008. 10.1002/cssc.202300008 36638158

[52] Shi C-Y, Zhang X-P, Zhang Q et al. Closed-loop chemically recyclable covalent adaptive networks derived from elementary sulfur. Chem Sci 2024; 15: 17460–8. 10.1039/D4SC05031B 39371464 PMC11447730

[53] Huang S, Shen Y, Bisoyi HK et al. Covalent adaptable liquid crystal networks enabled by reversible ring-opening cascades of cyclic disulfides. J Am Chem Soc 2021; 143: 12543–51. 10.1021/jacs.1c03661 34275290

[54] Deng Y, Zhang Q, Qu DH et al. A chemically recyclable crosslinked polymer network enabled by orthogonal dynamic covalent chemistry. Angew Chem Int Ed 2022; 61: e202209100. 10.1002/anie.202209100 PMC9804754 35922379

[55] Lu J, Wang H, Tian Z et al. Cryopolymerization of 1,2-dithiolanes for the facile and reversible grafting-from synthesis of protein–polydisulfide conjugates. J Am Chem Soc 2020; 142: 1217–21. 10.1021/jacs.9b12937 31927989

[56] Kariyawasam LS, Highmoore JF, Yang Y. Chemically recyclable dithioacetal polymers via reversible entropy-driven ring-opening polymerization. Angew Chem Int Ed 2023; 62: e202303039. 10.1002/anie.202303039 36988027

[57] Kassim AO, Kariyawasam LS, Yang Y. Degradable polydithioacetals with adjustable mechanical properties and insights into entropy-driven ring-opening polymerization. Macromolecules 2025; 58: 3395–406. 10.1021/acs.macromol.4c03237

[58] Smith RA, Fu G, McAteer O et al. Radical approach to thioester-containing polymers. J Am Chem Soc 2019; 141: 1446–51. 10.1021/jacs.8b12154 30636410

[59] Endo T, Bailey WJ. Synthesis and radical ring-opening polymerization of spiro o-carbonates. J Polym Sci, Polym Chem Ed 1975; 13: 2525–30. 10.1002/pol.1975.170131110

[60] Cho I . New ring-opening polymerizations for copolymers having controlled microstructures. Prog Polym Sci 2000; 25: 1043–87. 10.1016/S0079-6700(00)00022-8

[61] Watanabe H, Kamigaito M. Direct radical copolymerizations of thioamides to generate vinyl polymers with degradable thioether bonds in the backbones. J Am Chem Soc 2023; 145: 10948–53. 10.1021/jacs.3c01796 37079587 PMC10214439

[62] Elliss H, Dawson F, Nisa QU et al. Fully degradable polyacrylate networks from conventional radical polymerization enabled by thionolactone addition. Macromolecules 2022; 55: 6695–702. 10.1021/acs.macromol.2c01140

[63] Ivanchenko O, Destarac M. 1,1’- Thiocarbonyldiimidazole radical copolymerization for the preparation of degradable vinyl polymers. ACS Macro Lett 2023; 13: 47–51. 10.1021/acsmacrolett.3c00676 38118079

[64] Ivanchenko O, Mazières S, Mallet-Ladeira S et al. Radical copolymerization of thionoglycolide and derivatives for preparation of degradable vinyl polymers. Macromolecules 2024; 57: 8059–66. 10.1021/acs.macromol.4c01259

[65] Huang H, Wang W, Zhou Z et al. Radical ring-closing/ring-opening cascade polymerization. J Am Chem Soc 2019; 141: 12493–97. 10.1021/jacs.9b05568 31357865

[66] Sbordone F, Veskova J, Richardson B et al. Embedding peptides into synthetic polymers: radical ring-opening copolymerization of cyclic peptides. J Am Chem Soc 2023; 145: 6221–9. 10.1021/jacs.2c12517 36898136

[67] Laurel M, MacKinnon D, Becker J et al. Degradable thioester core-crosslinked star-shaped polymers. Polym Chem 2022; 13: 5579–89. 10.1039/D2PY00901C

[68] Chen Y, Wang R, Sheng X et al. Degradable and chain extendable segmented hyperbranched copolymers by wavelength-selective photoiniferter polymerization using a trithiocarbonate-derived dimethacrylate. ACS Macro Lett 2025; 14: 72–9. 10.1021/acsmacrolett.4c00771 39715460

[69] Bingham NM, Collins KE, Walsh J et al. Bespoke degradable polymers: modifying cyclic allyl sulfide lactones to tune polymer degradation rates and lower critical solution temperatures. Macromolecules 2025; 58: 516–25. 10.1021/acs.macromol.4c02681

[70] Jin Y, Hu C, Wang Z et al. Bio-based reprocessable and degradable epoxy resins via inverse vulcanization. ACS Sustain Chem Eng 2023; 11: 11259–68. 10.1021/acssuschemeng.3c02478

[71] Bao J, Martin KP, Cho E et al. On the mechanism of the inverse vulcanization of elemental sulfur: structural characterization of poly(sulfur-random-(1,3-diisopropenylbenzene)). J Am Chem Soc 2023; 145: 12386–97. 10.1021/jacs.3c03604 37224413

[72] Grimm AP, Plank M, Stihl A et al. Inverse vulcanization of activated norbornenyl esters—a versatile platform for functional sulfur polymers. Angew Chem Int Ed 2024; 63: e202411010. 10.1002/anie.202411010 38895894

[73] Yang H, Huang J, Song Y et al. Anionic hybrid copolymerization of sulfur with acrylate: strategy for synthesis of high-performance sulfur-based polymers. J Am Chem Soc 2023; 145: 14539–47. 10.1021/jacs.3c04746 37345976

[74] Arslan M, Akdeniz ŞG, Erdemi H. Polysulfide-ene polymerization of bisacrylamides and bismaleimides toward sulphur-rich polymers. J Macromol Sci A, Part A 2024; 61: 339–47. 10.1080/10601325.2024.2338382

[75] Tian T, Hu R, Tang BZ. Room temperature one-step conversion from elemental sulfur to functional polythioureas through catalyst-free multicomponent polymerizations. J Am Chem Soc 2018; 140: 6156–63. 10.1021/jacs.8b02886 29685036

[76] Chen L, Hu R, Tang BZ. Facile synthesis of functional polytrithiocarbonates from multicomponent tandem polymerizations of CS_2_, thiols, and alkyl halides. J Am Chem Soc 2025; 147: 1134–46. 10.1021/jacs.4c14708 39707976

[77] Silvano S, Carrozza CF, de Angelis AR et al. Synthesis of sulfur-rich polymers: copolymerization of cyclohexene sulfide and carbon disulfide using chromium complexes. Macromolecules 2020; 53: 8837–46. 10.1021/acs.macromol.0c01555

[78] Lei J-X, Wang Q-Y, Du F-S et al. Dynamic ring-chain equilibrium of nucleophilic thiol-yne “click” polyaddition for recyclable poly(dithioacetal)s. Chinese J Polym Sci 2021; 39: 1146–54. 10.1007/s10118-021-2587-y

[79] Watanabe S, Yano T, An Z et al. Aromatic poly(dithioacetal)s: spanning degradability, thermostability, and high refractive index towards eco-friendly optics. ChemSusChem 2024; 18: e202401609. 10.1002/cssc.202401609 39340202

[80] Du S, Yang S, Wang B et al. Acetal-thiol click-like reaction: facile and efficient synthesis of dynamic dithioacetals and recyclable polydithioacetals. Angew Chem Int Ed 2024; 63: e202405653. 10.1002/anie.202405653 38764409

[81] Sedrik R, Bonjour O, Laanesoo S et al. Chemically recyclable poly(*β*-thioether ester)s based on rigid spirocyclic ketal diols derived from citric acid. Biomacromolecules 2022; 23: 2685–96. 10.1021/acs.biomac.2c00452 35617050 PMC9198987

[82] Chen M, Duan S, Zhou L et al. Recyclable polymer-bonded explosives enabled by thiol-cured disulfide-based epoxy vitrimers. Polymer 2023; 276: 125949. 10.1016/j.polymer.2023.125949

[83] Kang X, Yu M, Zhao H et al. Pyridinyl polythioether via a one-pot thiolactone ring-opening and thiol-phenylsulfone click polymerization: synthesis, fluorescence, and degradation behavior. Macromolecules 2025; 58: 1878–87. 10.1021/acs.macromol.4c02963

[84] Hernandez JJ, Sama GR, Hu Y et al. Radical-mediated scission of thioaminals for on-demand construction-then-destruction of cross-linked polymer networks. Adv Funct Mater 2023; 34: 2306462. 10.1002/adfm.202306462

[85] Qian Y, Dong F, Guo L et al. Self-healing and reprocessable terpene polysiloxane-based poly(thiourethane-urethane) material with reversible thiourethane bonds. Biomacromolecules 2023; 24: 1184–93. 10.1021/acs.biomac.2c01230 36808988

[86] Kiker MT, Uddin A, Stevens LM et al. Onium photocages for visible-light-activated poly(thiourethane) synthesis and 3D printing. J Am Chem Soc 2024; 146: 19704–09. 10.1021/jacs.4c07220 38981090

[87] Chen K, Xie H, Liu J. Self-healing and mechanically robust poly(thiourea-disulfide) elastomers based on three types of synergistic dynamic bonding. Polym Chem 2024; 15: 2370–6. 10.1039/D4PY00322E

[88] Horiuchi S, Yamamoto D, Kaiho S et al. Well-controlled synthesis of poly (phenylene sulfide) (PPS) starting from cyclic oligomers. Macromol Symp 2015; 349: 9–20. 10.1002/masy.201300221

[89] Rivero-Crespo MA, Toupalas G, Morandi B. Preparation of recyclable and versatile porous poly(aryl thioether)s by reversible Pd-catalyzed C–S/C–S metathesis. J Am Chem Soc 2021; 143: 21331–9. 10.1021/jacs.1c09884 34871503 PMC8704200

[90] Liu J, Shi Y, Li J-J et al. Closed-loop recyclable vinylogous carbamothioate-based covalent adaptable networks. Macromolecules 2023; 56: 6644–54. 10.1021/acs.macromol.3c01142

[91] Aljuaid M, Chang Y, Haddleton DM et al. Thermoreversible [2 + 2] photodimers of monothiomaleimides and intrinsically recyclable covalent networks thereof. J Am Chem Soc 2024; 146: 19177–82. 10.1021/jacs.4c04193 38953610 PMC11258687

[92] Li W, Li Y, Song Z et al. PEDOT-based stretchable optoelectronic materials and devices for bioelectronic interfaces. Chem Soc Rev 2024; 53: 10575–603. 10.1039/D4CS00541D 39254255

[93] Muench S, Wild A, Friebe C et al. Polymer-based organic batteries. Chem Rev 2016; 116: 9438–84. 10.1021/acs.chemrev.6b00070 27479607

[94] Vanparijs N, Nuhn L, De Geest B. Transiently thermoresponsive polymers and their applications in biomedicine. Chem Soc Rev 2017; 46: 1193–239. 10.1039/C6CS00748A 28165097

[95] Rix MFI, Collins K, Higgs SJ et al. Insertion of degradable thioester linkages into styrene and methacrylate polymers: insights into the reactivity of thionolactones. Macromolecules 2023; 56: 9787–95. 10.1021/acs.macromol.3c01811

[96] Luzel B, Gil N, Désirée P et al. Development of an efficient thionolactone for radical ring-opening polymerization by a combined theoretical/experimental approach. J Am Chem Soc 2023; 145: 27437–49. 10.1021/jacs.3c08610 38059751

